# Droplet-Engineered Scalable High-Throughput Perovskite Micropatterning for Next-Generation Optoelectronics

**DOI:** 10.1021/acsnano.5c21710

**Published:** 2026-03-03

**Authors:** Bori Shi, Yubing Han, Mengying Zhang, Zhiyong Fan, Eugene A. Goodilin, Irina A. Veselova, Weijia Wen, Jinbo Wu

**Affiliations:** † Shenzhen Longgang District Key Laboratory of Power Battery Materials and Devices, Faculty of Materials Science, 508056 Shenzhen MSU-BIT University, Shenzhen 518100, China; ‡ Department of Electronic & Computer Engineering, The Hong Kong University of Science and Technology, Kowloon, Hong Kong 999077, China; § State Key Laboratory of Displays and Optoelectronics, The Hong Kong University of Science and Technology, Kowloon, Hong Kong 999077, China; ∥ SMBU-Shenzhen Hulk Micro-Nano Techn. Co., Ltd. Microfluidics Joint Lab, Faculty of Materials Science, Shenzhen MSU-BIT University, Shenzhen 518100, China; ⊥ Department of Materials Science, M. V. Lomonosov Moscow State University, Moscow 119991, Russian Federation; # Department of Chemistry, M. V. Lomonosov Moscow State University, Moscow 119991, Russian Federation; ¶ Advanced Materials Thrust, The Hong Kong University of Science and Technology (Guangzhou), Guangzhou 511453, China; ∇ Materials Genome Institute, Shanghai University, Shanghai 201900, China

**Keywords:** droplet array, high-throughput fabrication, solution processing, wettability, self-assembly, perovskite materials, patterning technology, optoelectronic devices

## Abstract

Droplet-based patterning techniques have demonstrated great effectiveness in fabricating highly ordered arrays for applications of functional materials in photonics, optoelectronics, and sensing technologies. Droplets act as small units and microreactors, providing controlled physical or chemical interactions, thereby enabling solution-processable material deposition on various substrates in a high-throughput, large-scale manner. Particularly for perovskite materials, droplet techniques allow the bottom-up fabrication of perovskite arrays, fully exploiting the cost reduction and efficiency improvement of solution-based processing for perovskite devices. This article aims to review the principles and recent progress in microdroplet array technology, explore its potential in perovskite material patterning, and discuss the broad applications of perovskite arrays in photonic and optoelectronic fields, including light-emitting diodes, photodetectors, fluorescent anticounterfeiting, and color conversion layers, to provide a comprehensive engineering reference for readers.

## Graphical abstract

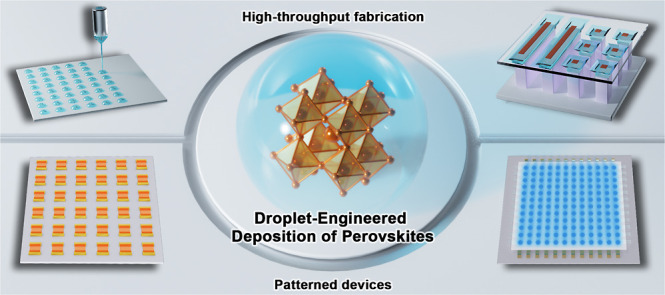

## 1 Introduction

As a flexible and efficient engineering tool, the droplet technology plays an increasingly important role in the fields of materials science, micropatterning, and nanotechnology. Recently, a lot of attention has been addressed to micropatterning of perovskite materials with excellent optoelectronic properties, demonstrating high absorption coefficients, long carrier diffusion lengths, low trap density, efficient photoluminescence performance and photovoltaic activity, tunable band gap characteristics, and emission spectral ranges.^1–3^ The solution processability of perovskite materials enables the full potential of droplet technology in precisely controlled self-assembly and patterning.^4^ With the trends of flexible, miniaturized, and arrayed preparation and device structure design, the excellent performance of perovskites combined with the ease of patterning and preparation has led to them being widely used in light-emitting diodes (LEDs), photodetectors, lasers, transistors, and memristors.^5–8^ Therefore, controlled growth or patterning of perovskite building blocks on ubiquitous silicon and other optoelectronic platforms is a critical step in the practical realization of advanced optoelectronic devices.

The development of micro- and nanofabrication methods is critical for the manufacture of perovskite optoelectronic devices with high-resolution patterning, alignment, and precise control of feature sizes.^9^ For example, the most traditional and accessible top-down technologies currently used in the semiconductor industry enable mask patterning via photolithography^10–13^ and electron beam lithography^14–19^ on perovskite polycrystalline films. However, owing to the inherent characteristics of perovskites, various ionic substances dissolve or degrade the solution when these substances encounter the polar solvents used in lithography, which severely reduces the quality and optical performance of the perovskite structure.^20^ Therefore, patterning perovskites via traditional photolithography is challenging. Researchers have used improved lithography methods to enhance the compatibility between materials and patterning techniques.^21^ For example, orthogonal lithography,^22–26^ lift-off patterning,^27,28^ two-step patterning,^29^ and photo-cross-linking patterning^30–34^ can be used to avoid damage to the perovskite film from subsequently utilized solvents. Maskless patterning techniques based on focused ion beam sputtering^35–37^ and laser ablation^38–41^ can directly segment perovskite crystal films, but these techniques inevitably cause chemical, photochemical, and thermal degradation during patterning,^42^ leading to surface damage and crystal structure defects, which may introduce significant optical loss into perovskite devices. In contrast, bottom-up patterning strategies can produce multiscale, multidimensional, and periodically micro/nanopatterned perovskites via different nondestructive pathways. For example, direct laser writing manufacturing technology can focus a beam on a target material to induce photothermal-effect-confined nucleation of perovskite crystals inside a transparent matrix or at an interface,^43–51^ photo-cross-link,^52^ and photon-effect-induced crystallization.^53^ Although the laser-induced crystallization method can achieve accurate patterning of perovskites, the difficulty of accessing and processing prepared crystal films makes it unfavorable for the subsequent construction of optoelectronic devices. In contrast, prepatterned topological, chemical, and crystallographic substrates fabricated via micro/nanoprocessing provide a convenient platform for template-assisted growth and graphoepitaxy of perovskite crystals. Through this construction strategy, the perovskite growth process can be precisely controlled to improve the crystal quality and device performance, and novel functions such as anisotropy and directional optoelectronic properties can be achieved.^54,55^ For example, pattern-selective epitaxial growth,^56–60^ surface-assisted vapor deposition,^20,61–64^ mold-embedded melt growth,^65^ and porous-structure-assisted vapor deposition^66–74^ have been performed. However, these methods usually require high vacuum and temperatures; therefore, they are not cost-effective. Therefore, there is a great demand for the development of alternative technologies for fabricating patterned perovskites, especially those based on nondestructive bottom-up technologies and low-cost solution-processed assembly technologies.

The microfluidic technology is based on the application of capillary forces, liquid surface tension, viscosity, and flow rate to achieve the generation, segmentation, arrangement, or redistribution of liquid flows and droplets.^75^ As an innovative high-throughput preparation method that significantly improves the preparation efficiency and controllability of solution-based materials, droplet array generation has gradually become the first choice for the construction of patterned perovskites. In recent years, by precise control of the evaporation environment and the size, spacing, and morphology of droplets, many researchers have developed perovskite arrays of various morphologies, such as quantum dots (QDs), nanowires, microsheets, and polycrystalline films. As illustrated in Figure 1, the “openness” of a microdroplet array makes the droplets easy to access and address from above the substrate, which facilitates the construction of miniaturized and arrayed perovskite photonic and optoelectronic devices. In this review, we aim to summarize the latest progress in the preparation of perovskite arrays via high-throughput droplet technology and investigate the generation mechanism of high-throughput microdroplet arrays, the bottom-up patterned growth methods for perovskites, and the droplet properties controlling several important aspects of perovskite crystallization. Moreover, examples of the application of low-temperature solution methods in the field of perovskite photoelectric device arrays, such as photodetector arrays and LED arrays, are presented for specific cases to demonstrate the role of microdroplet array technology in promoting technological innovations in the field of optoelectronics and its broad prospects. Although perovskite patterning reviews are available,^76^ few papers comprehensively discuss the perovskite patterning from the perspective of droplet array technology. More importantly, as a microdroplet is the smallest unit of an array of solution-based materials, we believe that a discussion of the development of microdroplet technology can also provide a valuable reference for arrays of other solution-based materials.

**Figure 1:**
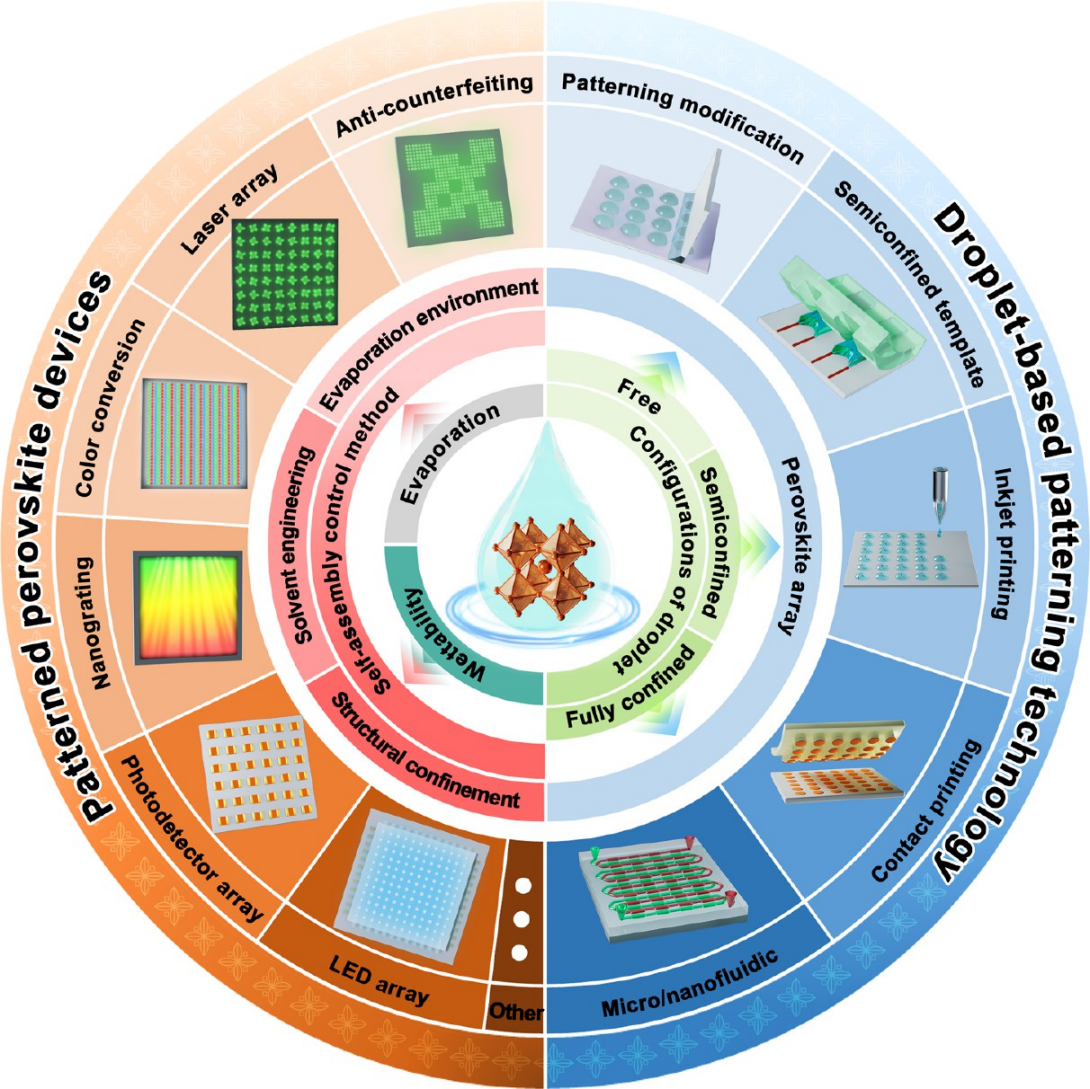
Overview of perovskite array fabrication based on the droplet method and its applications.

## 2 Fundamentals of Droplets

Droplets can simulate various conditions of the macroscopic reactors. Therefore, liquid droplets are often used as microreactors to achieve biochemical reactions, rapid mixing of reagents, and microparticle synthesis.^77,78^ With the development of microelectromechanical systems and soft lithography technology, microfluidic technology provides a means to precisely control very small volumes of fluids for chemical reactions and biological processes, enabling high-throughput experiments at the microscopic scale. Thus, material synthesis becomes possible, providing a powerful tool for the study and utilization of physicochemical phenomena on the microscale. In the field of material self-assembly, wetting and evaporation of liquids are two important processes. They directly determine the quality and interface characteristics of films and the morphology of crystals, thus affecting the performance of the final device.

### 2.1 Wettability of Droplets

Liquid wettability is critical for regulating microfluid flow to handle and manipulate microscale fluids.^79^ The contact angle θ is a measure of the static wettability on a solid surface and is determined by the force equilibrium at the solid–liquid–vapor three-phase contact line (TCL). For a droplet on a flat solid surface, the equilibrium contact angle is described by the famous Young’s equation:^80^

$$\cos\theta_{Y}=\frac{\gamma_{sv}-\gamma_{sl}}{\gamma_{lv}}$$

where θ_Y_ is the Young angle; γ is the interfacial tension; and the subscripts “s”, “l”, and “v” represent the solid, liquid, and gas phases, respectively. In general, the contact angle is >90° for a hydrophobic surface, whereas the contact angle is <90° for a hydrophilic surface. However, considering the physicochemical and structural states of water droplets, some researchers believe that the new limit between hydrophilicity and hydrophobicity of smooth solid surfaces may be a contact angle of 65°.^81^ In this work, the numerical limit is not strictly distinguished, and the focus is instead placed on whether surface modification is performed; e.g., a surface treated with plasma becomes more hydrophilic, whereas a surface treated with silane becomes more hydrophobic.

The dynamic contact angle is considered to characterize the dynamic wettability of a solid surface.^82^ Taking the tilt test of a sessile droplet as an example, for a tilt angle α less than the critical value, the droplet adheres to the surface and tilts along the tilt direction, making the contact angle at the advancing side larger than that at the receding side.^83–85^ This difference between contact angles due to surface roughness and/or chemical heterogeneity is referred to as contact angle hysteresis.^86,87^ When the critical tilt angle is reached, that is, when the gravity acting on the water droplet overcomes the lateral adhesion force, the fixed water droplet starts to slide.^88^ Similar to solid–solid friction, the lateral adhesion force between a droplet and a solid can be divided into two cases: static and dynamic. Once the adhesion force threshold is exceeded, the droplet transitions from the static state to the stable dynamic state.^89^ Therefore, the dynamic wettability of a droplet affects the spreading, retraction, and internal flow of the droplet, which have an important effect on the formation of droplet arrays on various microstructured substrates.

### 2.2 Evaporation Dynamics of Droplets

The volume of a droplet is usually in the range of picoliters to microliters, and the droplet inevitably evaporates and dries out.^90^ The evaporation of droplets is faster at the microdroplet scale.^91^ In the past 20 years, approximately 17,000 articles on droplets and evaporation have focused on the basic issue of droplet drying. Compared with the previous 20 years, the number of publications has increased by more than 10 times (based on Web of Science), and nearly 50% of these articles belong to the fields of materials and chemistry. The droplet deposition morphology has a major impact on printing, material self-assembly, and fabrication of solution-based devices.^92^

The three stages of sessile droplet evaporation on a nonhydrophobic substrate are shown in Figure 2a, where L_0_ represents the initial radius of the droplet and θ_0_ denotes the initial contact angle. In the first stage, the droplet base radius L is constant while the contact angle θ decreases for a long time; in the second stage, the contact angle θ is constant while the droplet bottom radius *L* decreases; and in the third stage, the radius *L* and contact angle θ at the droplet bottom both rapidly decrease until the droplet disappears.^93–95^ In this process, the evaporation of the solvent causes not only concentration of the solute but also spatial redistribution of the dispersed phase. The physical phenomena generated during the evaporation of droplets, such as the coffee ring effect, Marangoni flow, and surface trapping effect, significantly affect the flow and deposition of solute particles inside the droplet. Specifically, when the droplet dries on a solid surface, the pinning of the droplet contact line ensures that the liquid evaporated from the edge is replaced by the liquid from the inside, and the suspended particulate matter in the droplet flows from the center of the droplet to the edge via capillary flow.^96^ This phenomenon of sedimentation in a ring is referred to as the coffee ring effect.^97,98^ Moreover, during the evaporation process, the Marangoni effect, caused by the temperature gradient or concentration gradient at the liquid–air interface of the droplet, can form a recirculation flow.^99–101^ This flow causes the main position of solute deposition to be at the center of the droplet, which is opposite to the coffee ring effect.^102,103^ In addition, when the average interface descending rate (*V*_i_) exceeds the particle average diffusion rate (*X*_p_), the particles in the vertical evaporation flow will be trapped by the rapidly descending surface, forming a quasisolid layer. In particular, a low particle density and a high evaporation rate can increase the possibility of particles being trapped by the free surface.^104^ As shown in Figure 2b, the surface trapping effect makes the suspended particles tend to kinetically accumulate at the gas–liquid interface, which greatly prevents the particles from being delivered to the edge of the droplet, thus forming a more uniform deposition pattern.^105^

**Figure 2:**
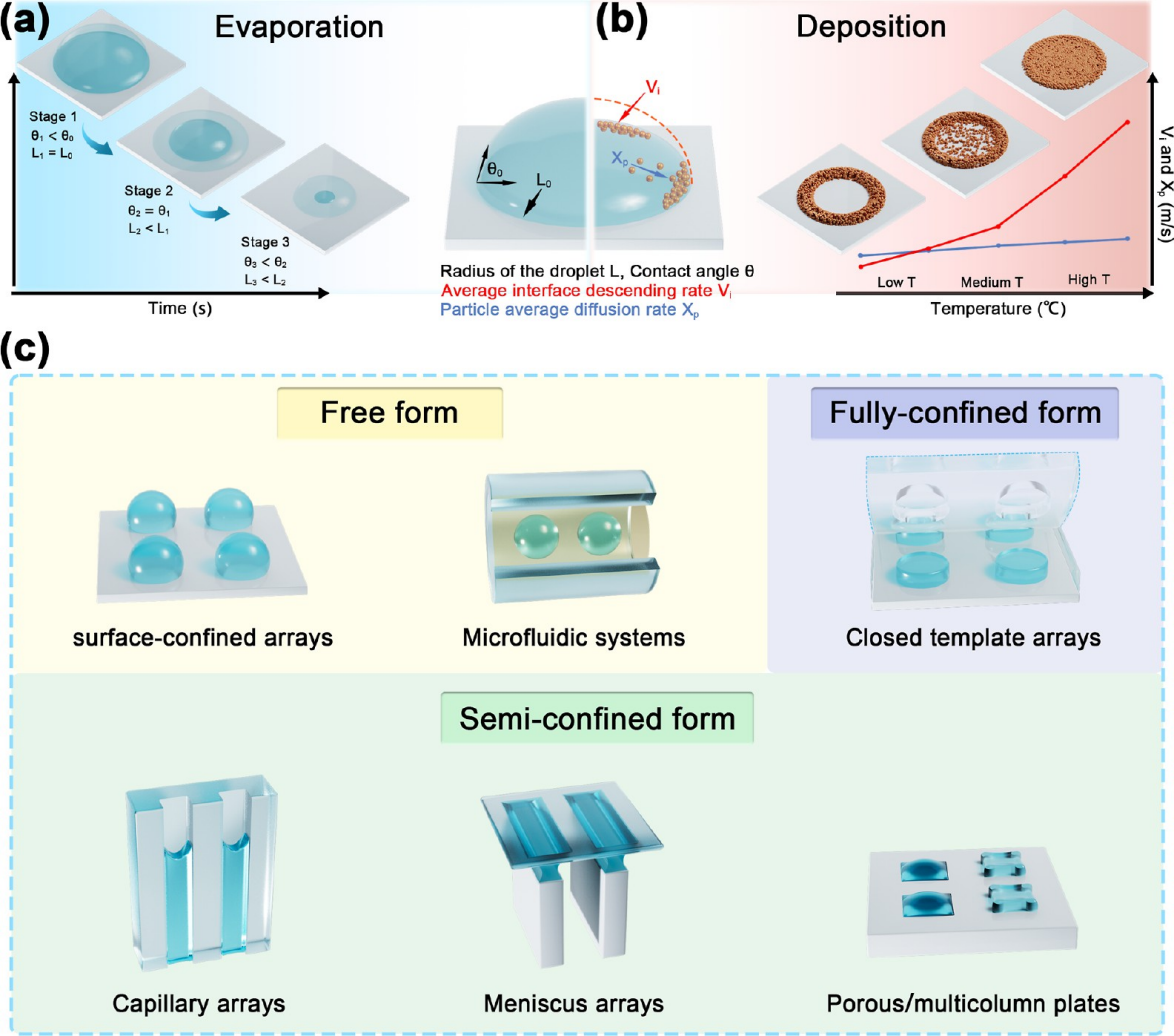
(a) Evaporation process of the droplet. (b) Deposition process of particles within the droplet. (c) Three forms of droplet arrays and representative generation methods.

On this basis, by changing the physical properties of the droplet (viscosity, surface tension, and volatility), substrate (surface material, thickness, thermal conductivity, wettability, and roughness), and surrounding environment (relative humidity, temperature, and pressure), the evaporation mode of the droplet and the behavior of the three-phase line can be regulated.^106–109^ Further study of this nonuniform redistribution process revealed that the particle flow inside the droplet (including capillary flow^110–112^ and Marangoni flow,^113–115^ surface trapping effect,^116,117^ and gravitational settling^118,119^ ), TCL dynamics,^120,121^ and particle shape^122,123^ affect the final particle distribution. Therefore, the evaporation process directly determines the morphology of the crystals obtained from perovskite precursors and the deposition patterns obtained from perovskite quantum dot dispersions. Specific cases are described and supplemented in the next section.

### 2.3 Configurations of Droplet Arrays

A droplet array refers to a number of droplets orderly arranged within a certain area, and each droplet unit is isolated from other units by space or surface barriers. (In this paper, independent liquid units are defined as droplets, which are not distinguished from rectangular liquid plugs.) The fabrication of droplet arrays depends on precise control of the fluid dynamics, template morphology, and wettability on solid substrates.^124^ The development of micro/nanoscale manufacturing and flexible device manufacturing technologies has made the preparation of droplet arrays more efficient.^125^ In general, the controllability of the liquid increases with decreasing free-fluid interface, but the use cost is relatively increased. As illustrated in Figure 2c, a liquid can be divided into three representative forms according to the degree of the free-fluid/fluid interface: the free form (droplet), semiconfined form (meniscus), and fully confined form (confined liquid).^126^

1) In a free-form droplet array, the liquid interface is completely unbound or partially constrained by a substrate to form sessile droplets. The main method to generate individual microsized droplets is to segment the liquid–liquid or air–liquid interface of the target liquid. For example, in microfluidic systems, the mechanism of droplet formation mainly depends on the fluid dynamics in microscale channels. Through stimuli such as squeezing, shearing, heating, magnetic field application, and sound field application in a microchannel, a discrete-phase fluid is divided into a series of free-phase fluids by a continuous phase (usually two mutually immiscible solutions, such as a water phase and an oil phase). This technology can be used to precisely control the transport, fusion, and splitting of droplets in the channel through programming. In contrast to microfluidic systems, surface-confined droplet arrays do not require pumps, valves, or tubes for droplet manipulation and avoid the clogging problem associated with complex channel networks.^127,128^ High-throughput arrays of mutually independent microdroplets can be generated on a substrate plane by using micro/nanoprocessing technology to construct heterogeneous wettability substrates and control the selective infiltration behavior of liquids on patterned surfaces.^129,130^ Liquids can wet and be pinned by hydrophilic patterns, whereas hydrophobic regions serve as boundaries to prevent liquid movement and merging.^131,132^ Owing to the wall-free design, this method can greatly increase the array density and allows researchers to directly access or manipulate droplets.^133^ Additionally, printing technology employing fluid dynamics, interfacial tension, acoustics, and electrohydrodynamic methods is used to achieve precise control of the droplet volume and distribution. This technique has the advantages of low consumption, automation, and high throughput and can form an ordered array of sessile droplets in the absence of physical barriers.^134^
2) For the semiconfined form of droplet arrays, the fluid–fluid interface is combined with two or more rigid surfaces. Fabrication techniques using semiconfined materials are essentially based on engineering the solid surface wetting behavior to construct liquid morphologies. Therefore, designing an appropriate template is critical.^135^ For example, the liquid surface tension effect can be incorporated into a stencil to generate a liquid bridge and a meniscus at the end of a capillary between two planar rigid bodies. The generated liquid bridge and meniscus can control the shape and flow of the liquid, thus achieving controllable and accurate assembly and patterning of particles under the confinement of the liquid. In addition, porous plates^124,136^ or multicolumn plates^137–139^ with exposed surfaces employ recessed or elevated structural boundaries to define discrete droplet regions. These sidewall interfaces function as physical partitions that inhibit droplet coalescence and material transfer while preserving morphological integrity. Such systems represent conventional implementations in biological, pharmaceutical, and chemical processes.
3) For the completely confined form of droplet arrays, the liquid is constrained by micro/nanosized discrete closed spaces, and the interface is surrounded by a solid surface. A template with a micro/nanostructure is designed via microfabrication technology, and each droplet unit is segmented into a specific shape and size via a stamp or imprinting to meet different experimental needs. Owing to the spatial confinement, this method plays an important role in precise control of chemical reactions, self-assembly of nanoparticles, high-throughput screening, and cell culture.

## 3 Strategies for Controlling Perovskite Crystallization in Droplet-Based Systems

Before we discuss perovskite patterning technology in depth, the common control strategies used in the self-assembly process must be briefly summarized. By understanding these key strategies, we can better understand the reasoning behind the subsequent discussion of patterning technology.

### 3.1 Evaporation Environment

The evaporation rate has a critical effect on the crystal growth and nucleation. First, the temperature and atmosphere can significantly affect physicochemical processes such as solute molecular diffusion, crystal nucleation, and crystal growth.^76^ For example, a difference in the evaporation rate inside a droplet leads to the so-called coffee ring effect, which affects the uniformity of the film.^143^ To eliminate this effect, a balance between outward capillary flow and inward Marangoni flow must be achieved.^144,145^ For the growth of perovskites, supersaturation of the solution is a necessary condition for perovskite nucleation. When the solute concentration in the solution is greater than the solubility of the perovskite, particle nucleation begins.^146^ As demonstrated in Figure 3a,b, lower temperatures (e.g., <25 °C) are conducive to the growth of perovskite single crystals;^141,147–149^ at higher temperatures (e.g., >60 °C), the rapid evaporation of the solvent accelerates the nucleation rate and formation of perovskite thin films.^116,140,150,151^ Higher temperatures usually help accelerate the crystallization process but may lead to an increase in the number of crystal defects. Therefore, researchers propose that rapid evaporation under vacuum or in a low-pressure environment helps prepare small-grain and low-dimensional perovskite films without the coffee ring effect.^152–155^

**Figure 3:**
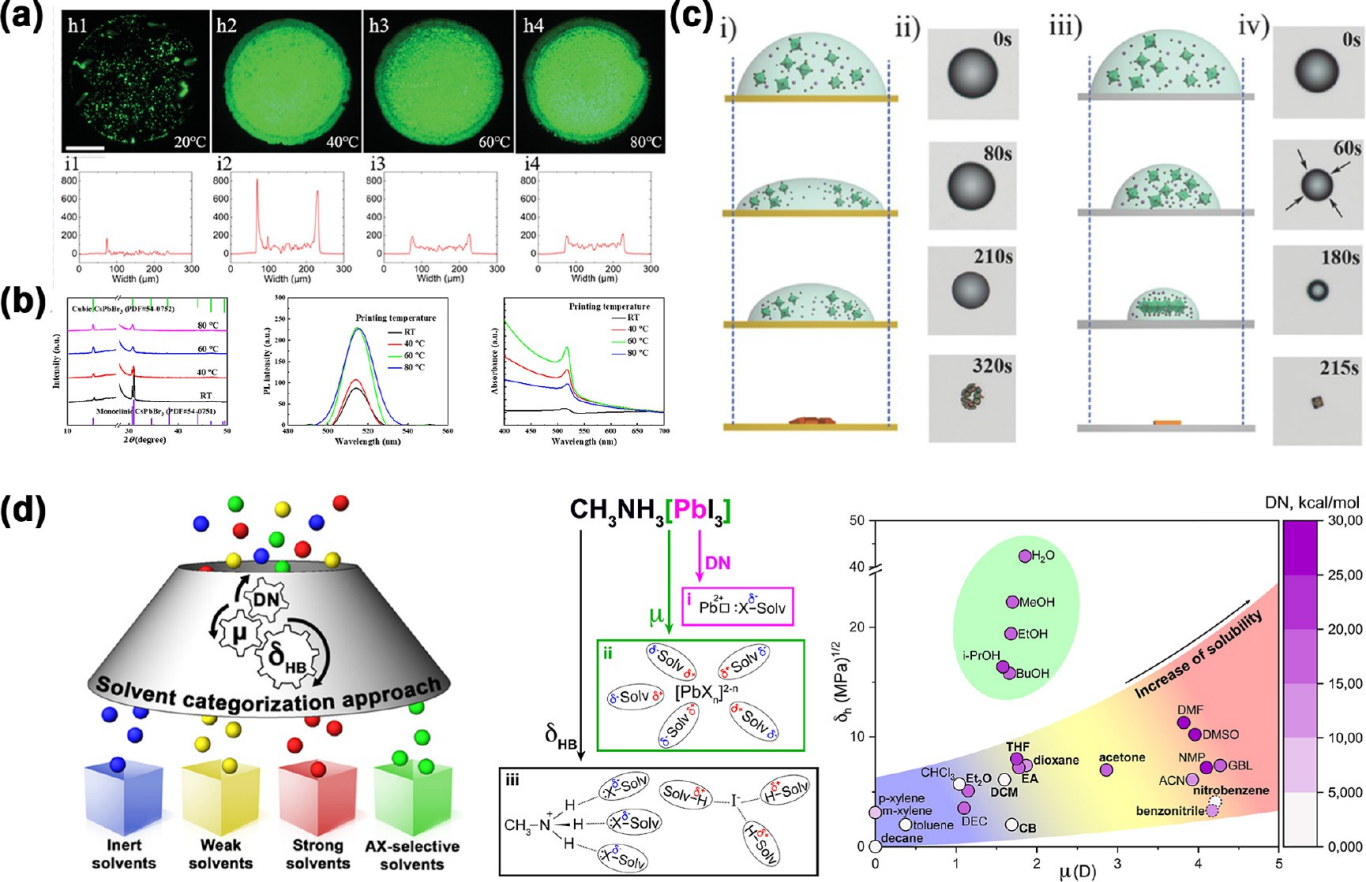
(a) Fluorescence images and surface profiles of perovskite films at different temperatures. (Reproduced with permission.^116^ Copyright 2021, Wiley-VCH.) (b) Normalized PL spectra and absorption spectra of the perovskite films printed at different temperatures. (Reproduced with permission.^140^ Copyright 2023, American Chemical Society.) (c) Schematic illustration and optical microscope images of the perovskite precursor droplet crystallizing on a high-adhesion substrate and low-adhesion substrate. (Reproduced with permission.^141^ Copyright 2016, Wiley-VCH.) (d) Schematic illustration of the main types of perovskite interactions with solvents. A set of solvents built in coordinates δHB−μ; DN values are presented in the form of a color map. (Reproduced with permission.^142^ Copyright 2020, American Chemical Society.)

Adhesion to the substrate is also a key factor in the formation of single-crystal perovskites. On high-adhesion substrates, the contact lines are pinned for a long time, and multiple crystals are prone to form in a droplet.^156^ As shown in Figure 3c, a low-adhesion substrate results in rapid retraction of the contact lines, facilitating orderly assembly and nucleation of perovskite molecules in the center of a droplet, forming a single crystal.^141^ Moreover, random nucleation leads to polycrystalline films or single-crystal films with random debris, and an unstable nucleus size is not conducive to controlling the film thickness. Perovskite seeds on a substrate can overcome the lattice mismatch and random nucleation barrier to facilitate epitaxial growth of perovskite crystals.^147,157,158^

The heat treatment method can not only significantly improve the uniformity of the film and device performance but also effectively eliminate the residual stress and internal defects, further optimizing the microstructure of the material. In such methods, the Ostwald ripening phenomenon, which is the process of dissolution of small particles and redeposition on the surface of large particles due to differences in particle solubility and size, is regarded as a key mechanism.^159^ Larger grains with lower surface energies further grow, whereas smaller grains disappear. This not only promotes homogenization of the particle distribution but also enhances the crystallinity and optoelectronic performance of the perovskite film.

### 3.2 Solvent and Additive Engineering

Solvent engineering has been proven to be important for controlling the nucleation and crystal growth of perovskites and for achieving uniformity and preventing pinholes.^160,161^ Supersaturation of the film through evaporation of the solvent in the precursor solution is a convenient and easy method to promote nucleation and growth of perovskite crystals,^142^ as illustrated in Figure 3d. Polar solvents such as *N*,*N*-dimethylformamide (DMF), dimethyl sulfoxide (DMSO), and γ-butyrolactone (GBL) have been widely reported.^162–165^ Cosolvents have relatively low solubility and vapor pressure compared to the main solvents, high surface tension, and favorable interactions with the main solvents. Early supersaturation, nucleation, and fast growth of single crystals occur through main solvent evaporation, while parasitic crystallization on the substrate is minimized. For example, the cosolvent evaporation strategy using *n*-cyclohexyl-2-pyrrolidone (CHP) effectively inhibits all competing phase transition pathways.^166^ Antisolvents (nonpolar solvents) have been widely used in the field of perovskite synthesis. The requirements are miscibility with the deposition solvent in the perovskite precursor solution and insolubility of the perovskite in the antisolvent. The solubility of a solute in a saturated perovskite solution is reduced by the addition of a miscible antisolvent, resulting in fast precipitation or fast crystallization.^167,168^ For example, antisolvents such as toluene, *n*-hexane, and ethyl acetate are not only used to prepare rod-shaped single crystals^149,169,170^ and bulk single crystals,^171^ but also often used to improve the flatness of thin films during the spin-coating process.^172–177^ Notably, the use of ligands in solvent engineering is equally important as they not only help reduce the structural damage to nanocrystals but also improve the stability of crystals by passivating the halogen vacancies on the surface. This step plays an important role in ensuring the integrity and long-term stability of crystals during self-assembly of perovskite droplets.^178–180^

Besides solvent engineering, researchers have also introduced additives to control the nucleation and crystal growth of perovskites. Researchers have used excess organic and lead components as chemical additives to control the crystallization kinetics.^181–183^ Physical additives such as high-viscosity polyvinylpyrrolidone (PVP) and the environmentally friendly ionic liquid methylammonium acetate (MAAc) can increase the viscosity of an ink and reduce the coffee ring effect caused by capillary flow.^184–189^ Researchers have introduced thermally durable poly­(vinyl alcohol) (PVA) and ultraviolet (UV)-*cross*-linkable acrylate polymers into ink to generate water-stable perovskite nanocrystal–polymer composites.^190–195^ Polyacrylate polymers can also be used to connect nanocrystals (NCs) to a polymer interface through surface grafting or copolymerization. The acrylate group undergoes radical polymerization under UV light, which cross-links the ligand with the adjacent perovskite NCs (PeNCs) to form a polymer network.^52,196,197^ Moreover, a hybrid hydrophobic polymer, such as polystyrene (PS), can further protect PeNCs from ambient water vapor.^198^ This polymer barrier effect, combined with solvent engineering and the use of additives, jointly improves the comprehensive performance of the perovskite droplet self-assembly products. Beyond organic substances, inorganic substances such as silica,^199–203^ titanium dioxide,^204^ zirconia (ZrO_2_),^205^ and ZnS^206,207^ can also be used to limit the growth size of perovskite crystals.^208^ Perovskite quantum dots encapsulated in inorganic particles exhibit better oxidation resistance, humidity resistance, and luminescence performance.^209^ Additives are not limited by the examples presented above. This article will not describe this in detail.

### 3.3 Structural Confinement Engineering

The physical three-dimensional structure and size of a droplet affect its evaporation and self-assembly. Accurate design of the liquid structure provides precise control of the growth of low-dimensional perovskite single crystals.^4^ For example, in a microchannel array, each microchannel can be considered a thin capillary tube, as shown in Figure 4a. Each microchannel helps guide spontaneous wetting of the precursor solution along the channel to form perovskite micro/nanowires^210–217^ and heterostructures.^218–220^ The wettability-mediated micropillar array ensures the formation of a liquid meniscus by splitting the precursor liquid film and anchoring regular microdomains above the micropillars.^221,222^ As shown in Figure 4b,c, nucleation and growth of perovskites are confined to these microdomains, resulting in square single-crystal perovskite microplates of uniform size and precise positioning.^223,224^ Other researchers have formed 3D perovskites with definable shapes through free-form guidance of perovskite crystallization driven by evaporation of the precursor meniscus.^225–227^ Additionally, during crystal growth, the isotropic growth conditions can be broken, and the growth of a perovskite into a sheet-like single crystal can be limited by pressure control through cover plates with microscale gaps.^57,228–232^

**Figure 4:**
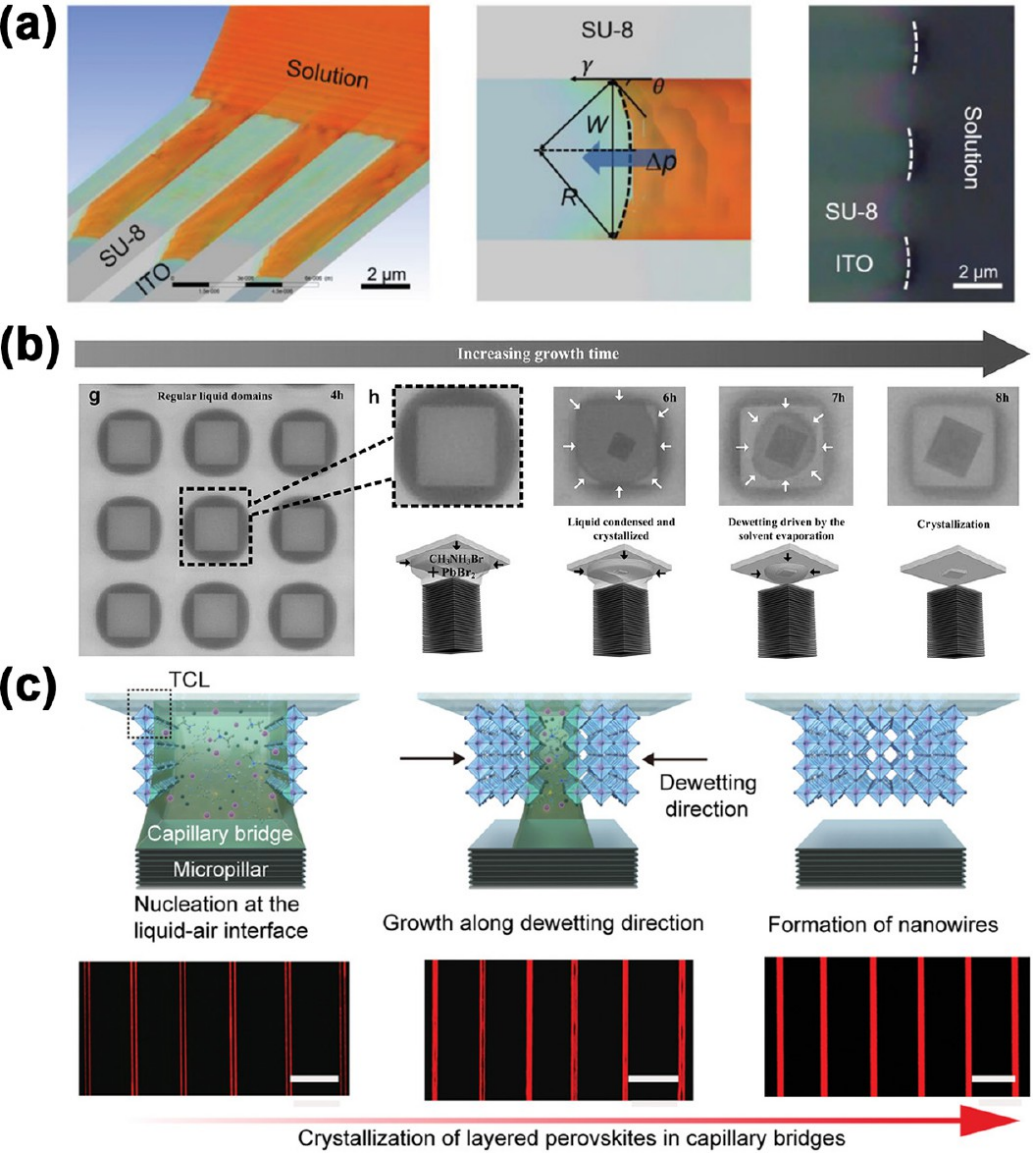
(a) Mechanism for microchannel-confined crystal growth. (Reproduced with permission.^211^ Copyright 2020, Wiley-VCH.) (b) Controllable dewetting process by the “liquid knife”. (Reproduced with permission.^223^ Copyright 2016, Wiley-VCH.) (c) Schematic illustration of the crystallization of layered perovskite nanowires in a capillary bridge. (Reproduced with permission.^224^ Copyright 2020, Wiley-VCH.)

Macroscopic arrangement of single-crystal arrays can be achieved using structured templates.^223,233,234^ However, the inherent randomness of nucleation results in randomness of the deposition position and direction of the crystals in each constrained unit, even if the array arrangement is macroscopically satisfied. Moreover, whether the template has a microwell structure or a surface-patterned structure, the random distribution phenomenon is not conducive to further integration of devices.^149,235–237^ Some researchers have proposed a gravity-mediated-assisted self-alignment method for the precise assembly of perovskite single crystals. By aligning a wettability-mediated square prism array, the crystal nuclei in a droplet gradually move to the bottom of the suspended droplet under the action of gravity. When a crystal grows to a certain size, it undergoes motion and rotation due to the comprehensive influence of its own gravity and the surface tension of the droplet, achieving precise alignment,^238^ as captured in Figure 5a. Moreover, the pressure gradient caused by wetting on the asymmetric surface of a topographic template has also been reported to guide the local growth of perovskites and achieve a sublithographic resolution of <50 nm. The principle is to use the contact angle between the precursor solution and the pore sidewall as well as the pore geometry to regulate the meniscus shape. As shown in Figure 5b, in a triangular microwell structure, the asymmetric meniscus can form a directional pressure gradient, allowing precise placement of NCs at specific locations.^239,240^

**Figure 5:**
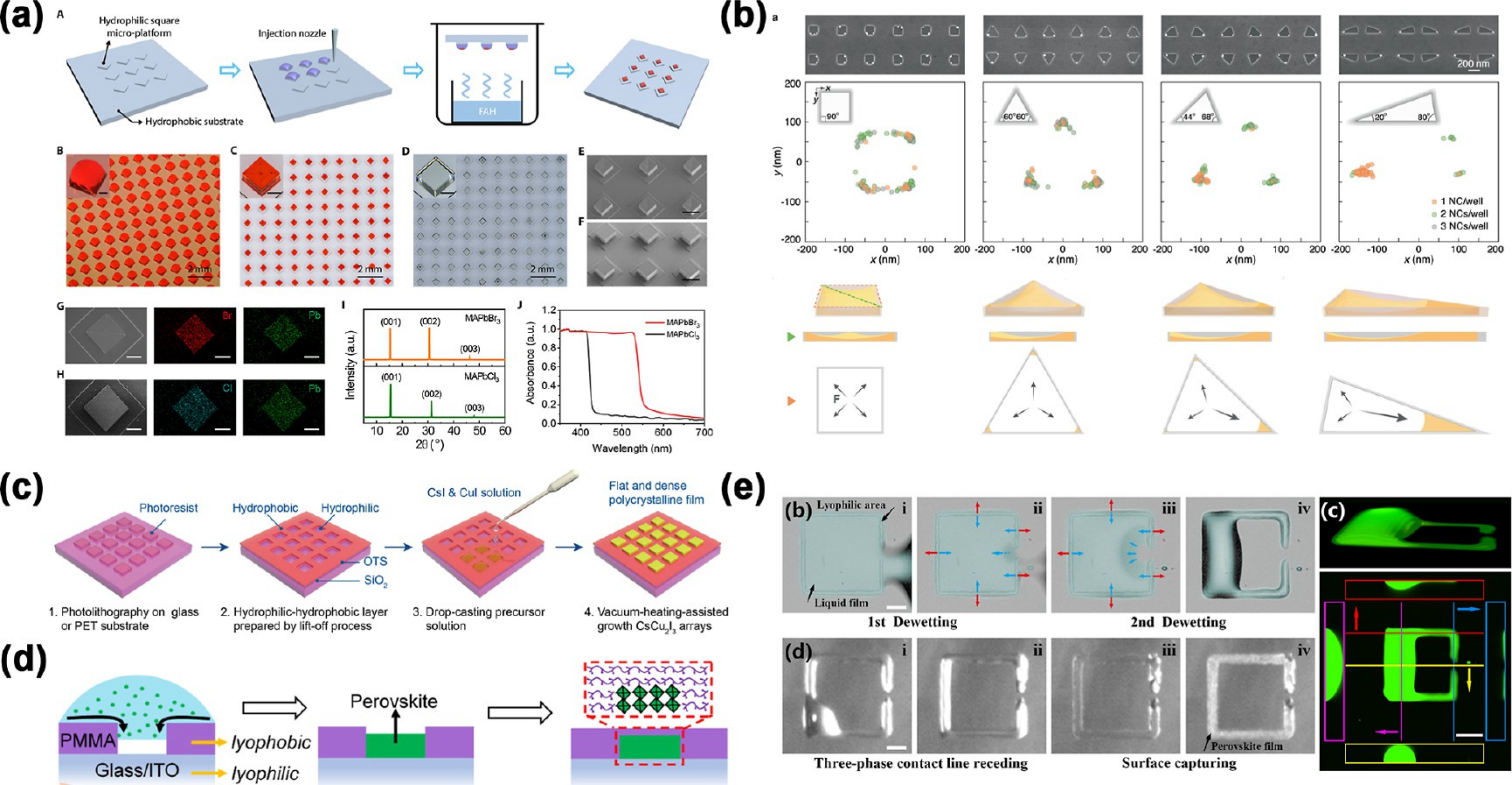
(a) Schematic diagram of the preparation process of perovskite single-crystal arrays and stereomicroscope images of the dyed red square precursor droplets. (Reproduced with permission.^238^ Copyright 2024, The American Association for the Advancement of Science.) (b) Deterministic nanocrystal placement with asymmetric meniscus. (Reproduced with permission.^239^ Copyright 2023, The Authors, published by Springer Nature.) (c) Schematic illustration of the fabrication procedure for the controlled growth of the patterned CsCu_2_I_3_ film. (Reproduced with permission.^241^ Copyright 2023, Wiley-VCH.) (d) Schematic illustration of the wetting–dewetting process that determines the nucleation rate of the perovskite. The function of the PMMA layer, which acts as a mask to protect the prepatterned arrays in the next overprinting procedures. (Reproduced with permission.^242^ Copyright 2022, American Chemical Society.) (e) The dewetting and evaporation processes of split-ring structured perovskite precursor solution captured by a high-speed camera. (Reproduced with permission.^117^ Copyright 2022, The Authors, published by Springer Nature.)

For perovskite polycrystalline films, poor crystallographic density will cause defects and current leakage, which seriously affects the optoelectronic performance of devices.^243^ The core idea of structural engineering is to allow more volumes of the precursor to crystallize within a smaller area, thereby improving the crystallization efficiency of the precursor. Researchers have used a wettability-assisted microwell structure as a container for storing a precursor liquid. As illustrated in Figure 5c,d, the lyophilic bottom surface and lyophobic well wall allow the precursor perovskite to be deposited only in the microwell structure, while there are no crystals in the hydrophobic area.^241,242^ Additionally, some researchers have proposed that through the design of lyophilic/lyophobic patterns, the precursor solution can be delivered to smaller lyophilic areas for deposition. As demonstrated in Figure 5e, the split ring structure can spontaneously undergo a second dewetting during evaporation, which can reduce the deposition area by more than 60% and increase the compactness of the crystal film when the ability to capture the solution remains the same.^117^

## 4 Fabrication of Perovskite Arrays

In this article, advanced strategies for preparing perovskite arrays are discussed in detail based on the classification of high-throughput droplet generation technology. Specifically, microfluidic, inkjet printing, transfer printing, and surface confinement techniques can form free-form droplet arrays; capillary force or external force-mediated template semi-confinement approaches are used to construct semiconfined droplet arrays; and fully constrained droplet arrays are usually realized through template confinement methods and nanoimprinting methods. These techniques not only demonstrate high precision and flexibility in droplet manipulation but also indicate future directions for the design and functionalization of perovskite materials. Through in-depth analysis of the principles and applications of these technologies, this article aims to help readers better understand the close relationship between the droplet generation technology and perovskite preparation, thus providing valuable guidance for further optimization of the performance of perovskite devices.

### 4.1 Patterned Wettability-Assisted Technology

Surface modification is a method of changing the surface characteristics of a material through chemical or physical means that provides the basis for surface hydrophilic or hydrophobic treatment. The behavior of liquids on a surface, for example, the wetting and evaporation behavior and even the fusion, bouncing, rotation, and splitting behavior of liquid droplets, is accurately regulated through precise control of the hydrophilicity and hydrophobicity of different regions on the material surface.^244–250^ Among the surface modification methods, the fabrication of wettability-patterned matrices depends on the precise control of the surface chemistry and topography, and patterning of hydrophilic or hydrophobic regions on solid substrates has become an advanced form of surface modification.^251^

#### 4.1.1 Surface Confinement

Through discontinuous dewetting on a lyophilic and repellent patterned surface, a liquid can be broken up into a series of isolated microdroplets with complex geometries,^252–257^ as illustrated in Figure 6a,b. The process underlying the generation of a 2D microdroplet array on a prepatterned plane can be considered the combination of a microtiter plate and microarray technology.^258^ External force-assisted dewetting combined with surface confinement techniques has emerged as a common approach for high-throughput droplet generation. Typical methods include spin coating, slide coating, confined coating, and dip coating on hydrophilic–hydrophobic patterned substrates, as illustrated in Figure 6b. In the preparation of perovskite arrays, no additional microstructures are required on the substrate surface; therefore, the open design of the preparation system makes the perovskite easy to access and integrate, thus improving the flexibility and efficiency of operation.

**Figure 6:**
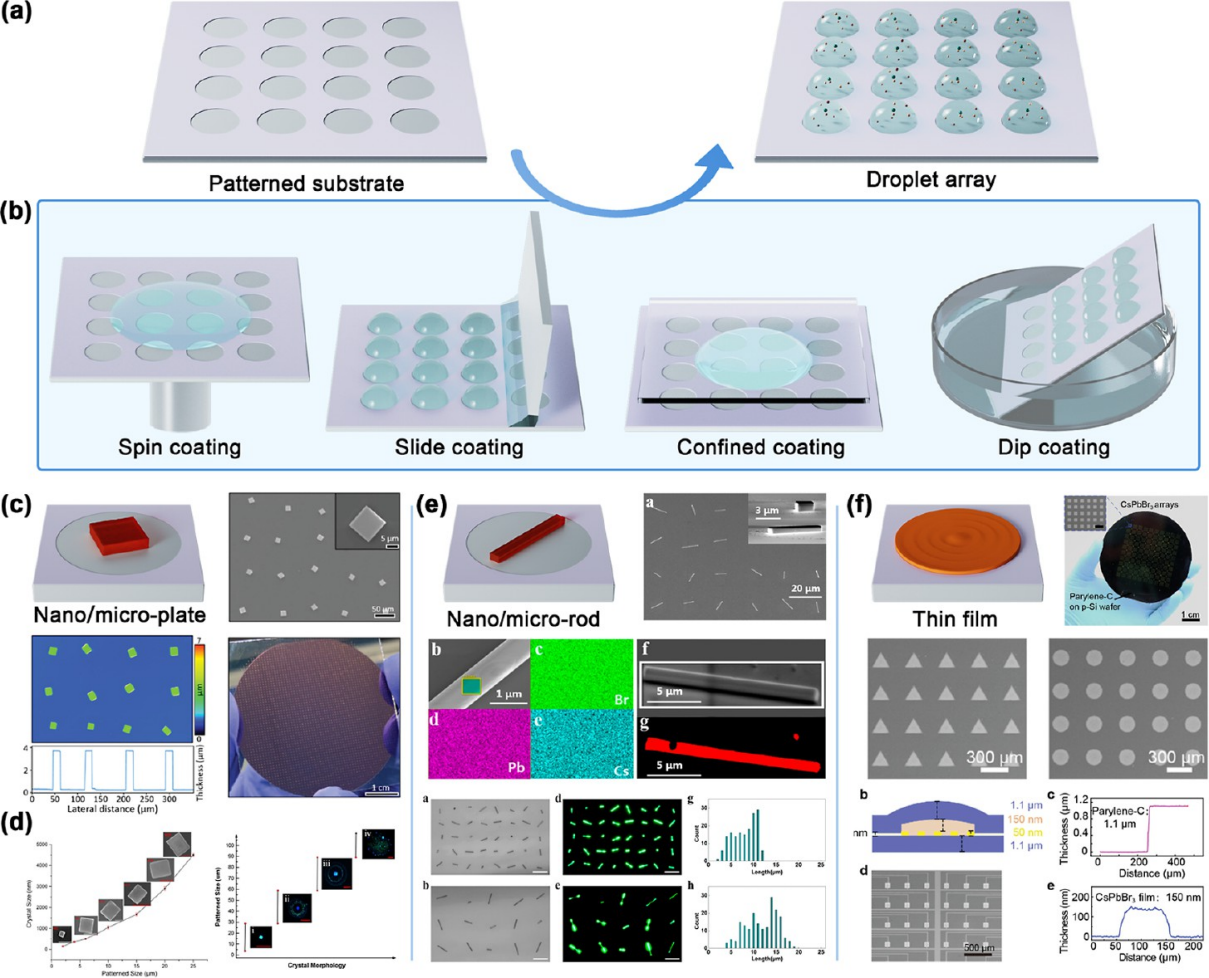
(a) Schematic illustration of droplet array generation on a patterned surface. (b) Several common methods for generating droplet arrays. (c) Microscopic image and 3D surface topography image of the patterned CH_3_NH_3_PbBr_3_ microplates. (Reproduced with permission.^235^ Copyright 2022, Wiley-VCH.) (d) Dependence of the crystal width on the concentration of the precursor and the patterned size. The fluorescent micrograph presents representative morphology changes of a perovskite crystal after surface droplets of different patterned sizes evaporated. (Reproduced with permission.^148^ Copyright 2020, American Chemical Society.) (e) Morphology and crystal structure of CsPbBr_3_ nanorod arrays. Bright-field and fluorescence micrographs and size distribution of different-sized CsPbBr_3_ nanorod arrays. (Reproduced with permission.^149^ Copyright 2021, American Chemical Society.) (f) The thickness of the ultrathin parylene-C film and the CsPbBr_3_ films. Photograph of large-scale CsPbBr_3_ arrays grown on a 4 in. p-Si wafer covered with parylene-C film. (Reproduced with permission.^152^ Copyright 2021, Wiley-VCH.)

Wu’s team used the discontinuous dewetting strategy to generate high-throughput perovskite precursor droplet arrays.^117^ By customizing the lyophilic pattern and adjusting the evaporation environment and the solvent atmosphere, the growth of target perovskites with different compositions, morphologies, and sizes was achieved.^116,148,149,259,260^ The substrates were modified to be lyophobic using 1H,1H,2H,2H-perfluorooctyltriethoxysilane (POTS), followed by photolithography to create periodic lyophilic arrays. These arrays can effectively guide the dewetting process and immobilize, split, and confine the TCL of the perovskite precursor in the lyophilic regions, forming tens of thousands of isolated perovskite precursor droplets. As shown in Figure 6d, the evaporation mode and internal flow field of the precursor droplets are controlled by adjusting the evaporation rate, and the lateral size and thickness of the single-crystal perovskite plates are regulated by adjusting the volume of the precursor solution.^148^ This preparation system was placed in a solvent atmosphere, and a one-step recrystallization was used to synthesize single-crystal perovskite nanorod arrays with a controllable morphology. The overall size distribution range of the nanorods, as demonstrated in Figure 6e, can be flexibly adjusted by forming patterns of different sizes on the substrate.^149^ Moreover, low-cost nanosecond laser ablation technology can be used to directly prepare lyophilic pattern arrays on lyophobic surfaces and generate unique textures on substrate surfaces. This texture enhances the randomness of nucleation, which is conducive to the generation of unclonable characteristics. For example, the CsPbCl_*x*_Br_3‑*x*_ perovskite crystal array generated by component segregation had random multiwavelength emission,^259^ and the CsPbBr_3_ nanopolycrystalline thin film^116,260^ had a specific outer profile shape and a unique microscopic texture. Shi et al. further developed a dual-functional laser ablation strategy for the one-step fabrication of patterned lyophilic surfaces and electrode arrays. The energy at the center of the Gaussian laser beam was sufficient to etch the indium tin oxide (ITO) electrode layer. Moreover, the thermal effect generated by the edge of the laser spot vaporized the lyophobic POTS layer. Since the width of the heat-affected zone was always significantly larger than the width of the etching zone, the area that could be wetted by the precursor solution could completely cover the ITO trench. The split-ring lyophilic pattern design enabled directed transport of the precursor solution to both sides of the ITO channel, which increased the density of the perovskite crystal film and provided new insights for preparing lateral structure devices.^117^ Pan’s team demonstrated the formation of perovskite crystal film arrays with defined geometries and sizes on a hydrophilic–hydrophobic patterned surface.^152,241,261–263^ For example, Wu et al. used a mixed solution of hexane and octadecyltrichlorosilane (OTS) to obtain a substrate with lyophobic properties. The substrate with a patterned mask formed after photolithography was then treated with phosphoric acid solution and oxygen plasma to obtain a surface with a periodic lyophilic pattern array. He used a mixed solution of PbI_2_ and PbCl_2_ in DMF as the first-step precursor droplets and then spin-coated it with a CH_3_NH_3_I precursor solution to convert the PbI_2_/PbCl_2_ array into a CH_3_NH_3_PbI_3–x_Cl_*x*_ array.^261,262^ On the basis of this patterning method, solvent evaporation was accelerated by vacuum-assisted deposition, and a pure inorganic CsPbBr_3_ perovskite^152^ and lead-free CsCu_2_I_3_ thin film arrays^241^ with good crystal uniformity and consistency were prepared, as shown in Figure 6f. Liang et al. sputtered a SiO_2_ layer on a substrate with a prepatterned bottom electrode and used 1H,1H,2H,2H-perfluorodecyltrimethoxysilane (FAS17) to form a hydrophobic surface. During droplet patterning, an antisolvent ethyl acetate solution was added dropwise to promote dense deposition of the perovskite crystal film. This strategy can be applied to a variety of different material systems and is fully compatible with existing lithography and etching technologies.^263^

Apart from using silane compounds to lyophobically modify substrates,^264,265^ researchers are also committed to developing other micro/nanoprocessing techniques that enable lyophilic patterning. For example, Wu et al. developed Cs-doped FAPbI_3_ perovskite thin films via graphene-assisted hydrophilic–hydrophobic surface-induced growth. First, by transferring a chemical vapor deposition (CVD)-grown graphene film onto a substrate, a micropattern was defined via photolithography, and then the exposed graphene was selectively removed via oxygen plasma treatment to form hydrophilic regions. Next, a perovskite precursor solution was spin-coated on the substrate, and the wetting/dewetting behavior of the solution on the hydrophilic/hydrophobic regions was used to induce the localized generation of droplet arrays. Large-scale patterned growth of perovskite thin films was realized after thermal annealing.^266^ Wang et al. used a polymer stamp combined with an imprinting technique to prepare a predesigned periodic hydrophilic/hydrophobic substrate to assist the patterned growth of CH_3_NH_3_PbX_3_ (X = Cl, Br, I) perovskite thin film arrays. Polydimethylsiloxane (PDMS) stamps with predesigned patterns were fabricated via UV lithography and replication molding techniques. A lyophobic pattern was formed because of the migration of the uncured oligomers in the PDMS stamp in close contact with the substrate. During the spin-coating process, the hydrophilic areas were wetted by the perovskite precursor solution, whereas the excess solution in the hydrophobic areas was removed from the substrate by the centrifugal force due to the dewetting properties. After thermal annealing, a perovskite film with the desired micropattern was formed on the substrate.^267^

#### 4.1.2 Microstructure Confinement

Based on the wettability-assisted patterning technique, the introduction of a physical microstructure can further improve the accuracy of the perovskite pattern. A physical template can place effective geometrical constraints on the perovskite solution so that perovskite arrays with more uniform shapes and sizes can be prepared to meet the integration requirements.^268^ Wu et al. patterned hydrophobic poly­(4-butylphenyldiphenylamine) (poly-TPD) on a substrate via photolithography, producing a microwell structure pattern. A patterned CH_3_NH_3_PbI_3_ perovskite film was then deposited via a one-step solution process. Finally, chlorobenzene (CB) was drop-coated to remove the poly-TPD layer, thus achieving a nonporous hybrid perovskite thin film array with an arbitrary micropattern.^269^ Another study showed that the CH_3_NH_3_PbBr_3_ precursor solution could be filled into the poly-TPD microwell structure via a blade coating process. The sample was then placed in an atmosphere of the antisolvent isopropanol (IPA). According to the Ostwald ripening theory, smaller crystals dissolved and were redeposited on larger crystals. Finally, unwanted poly-TPD was washed away with CB to form a CH_3_NH_3_PbBr_3_ single-crystal microplate array.^234^

Easy-to-pattern poly­(methyl methacrylate) (PMMA) microplates have also been reported for the preparation of perovskite arrays.^237^ Wang et al., under the dual effects of wettability and PMMA-template-limited crystallization, fabricated large-scale patterned arrays of perovskite microstructures with a controllable geometry and position. First, PMMA was spin-coated onto the substrate as a patterned resist. A circular microstructure array was subsequently constructed on the PMMA film via electron beam etching. PMMA is lyophobic, whereas the substrate is lyophilic, causing differences in the internal and external wettabilities of the template. The spontaneous wetting/dewetting behavior of the precursor on the template induced nucleation and growth of MAPbX_3_ perovskite in predefined circular pores.^270,271^ After the perovskite pattern was prepared, another PMMA resist layer was spin-coated on the first patterned array as a mask to prevent cross-contamination by the solvent. A full-color CsPbX_3_ perovskite microdisk thin film array was obtained by overprinting with different precursor solutions multiple times.^242^

#### 4.1.3 Cover Plate Confinement

The surface-confined droplet patterning technique involves an open surface and a planar substrate, and the growth space of perovskite droplets is limited by a lyophobic cover plate, thereby enabling the accurate generation of single-crystal microplate arrays. Zhang et al. reported strategies for inhibiting multiple nucleation to control the nucleation and growth processes of high-quality single-crystal perovskite microplate arrays with a uniform morphology. By combining selective sputter deposition and trichloro­(1H,1H,2H,2H-perfluorooctyl)­silane (FOTS) vapor modification techniques, through regulation of the wettability of patterned Au nanoparticles (NP) on the substrate, subtle adjustment and strict regulation of the nucleation energy barrier significantly suppressed random and multiple nucleation of perovskite crystals during the traditional wettability patterning process. In addition, the nucleation density of the perovskite crystals on the Au NP film was significantly reduced by placing a hydrophobic substrate on the solution to reduce the evaporation rate in a microsized confined space. As shown in Figure 6c, the crystallization of perovskite crystals on desired regions of Au NP thin films achieved precise control of the growth positions and improved the crystal uniformity.^235^ Wang et al. printed poly­(acrylic acid) (PAA) dissolved in a water/ethylene glycol mixture on quartz glass and used it as a polymer mask. The quartz surface was then hydrophobically functionalized with 1H,1H,2H,2H-perfluorodecyltrimethoxysilane (PFOS). Finally, the substrate was washed with ethanol to remove the PAA array, leaving a hydrophilic/hydrophobic patterned substrate. The perovskite precursor solution was then dropped on the pretreated substrate and covered with a hydrophobic quartz glass. As the solvent evaporated, a single-crystal MAPbCl_3_ perovskite array with precise positions and a uniform size was fabricated.^272^ Xu et al. sputtered SiO_2_ onto a substrate with a photoresist pattern and then soaked it in a mixed solution of *n*-hexane and OTS for hydrophobic modification. The photoresist was then stripped with acetone to form a hydrophilic/hydrophobic pattern. Finally, steric confinement by a hydrophobic glass cover plate and an antisolvent atmosphere to assist perovskite crystallization was realized, and on-chip fabrication of large-scale single-crystal MAPbBr_*x*_Cl_3‑*x*_ perovskite arrays was realized.^236^

### 4.2 Semiconfined Template-Assisted Technology

Template-assisted methods are techniques in which the size and arrangement of droplets are precisely controlled through physical space limitations and can be applied to a variety of low-viscosity material systems, including solutions such as perovskite precursor and quantum dot solutions, and for polymer, silicon wafer, glass, and metal substrates. Compared with the surface-confined method, the template-assisted method provides greater spatial control accuracy and is conducive to controlling the evaporation environment of the perovskite precursor.

#### 4.2.1 Capillary Force-Driven Template-Assisted Growth

When a liquid is in contact with a solid, the meniscus refers to the curved liquid surface formed by the cohesion force between liquid molecules and the adhesion force between the liquid and the solid.^273,274^ An immersion-type nanoparticle dispersion in a capillary is taken as an example as illustrated in Figure 7a. Surface tension will cause the liquid under the concave meniscus to experience additional pressure, which will drive the liquid to rise along the capillary wall. As evaporation progresses, the meniscus formed at the end is continuously pushed inward by the liquid–gas interface, and nanoparticles assemble one dimensionally along the channel.^275–277^ At the microscopic scale, the capillary effect is particularly important for microfluidic devices because of their high surface area-to-volume ratio.^278,279^ In periodic microchannels fabricated by soft lithography, spontaneous filling of the perovskite precursor is driven by the capillary force generated during contact with the substrate, producing aligned perovskite nanowire arrays.^280–282^ Owing to the semiclosed design of the template, introducing the system into an antisolvent atmosphere for growth is convenient, and the nanowires will have a smoother surface and fewer crystal defects through control of the crystallization kinetics.^283^

**Figure 7:**
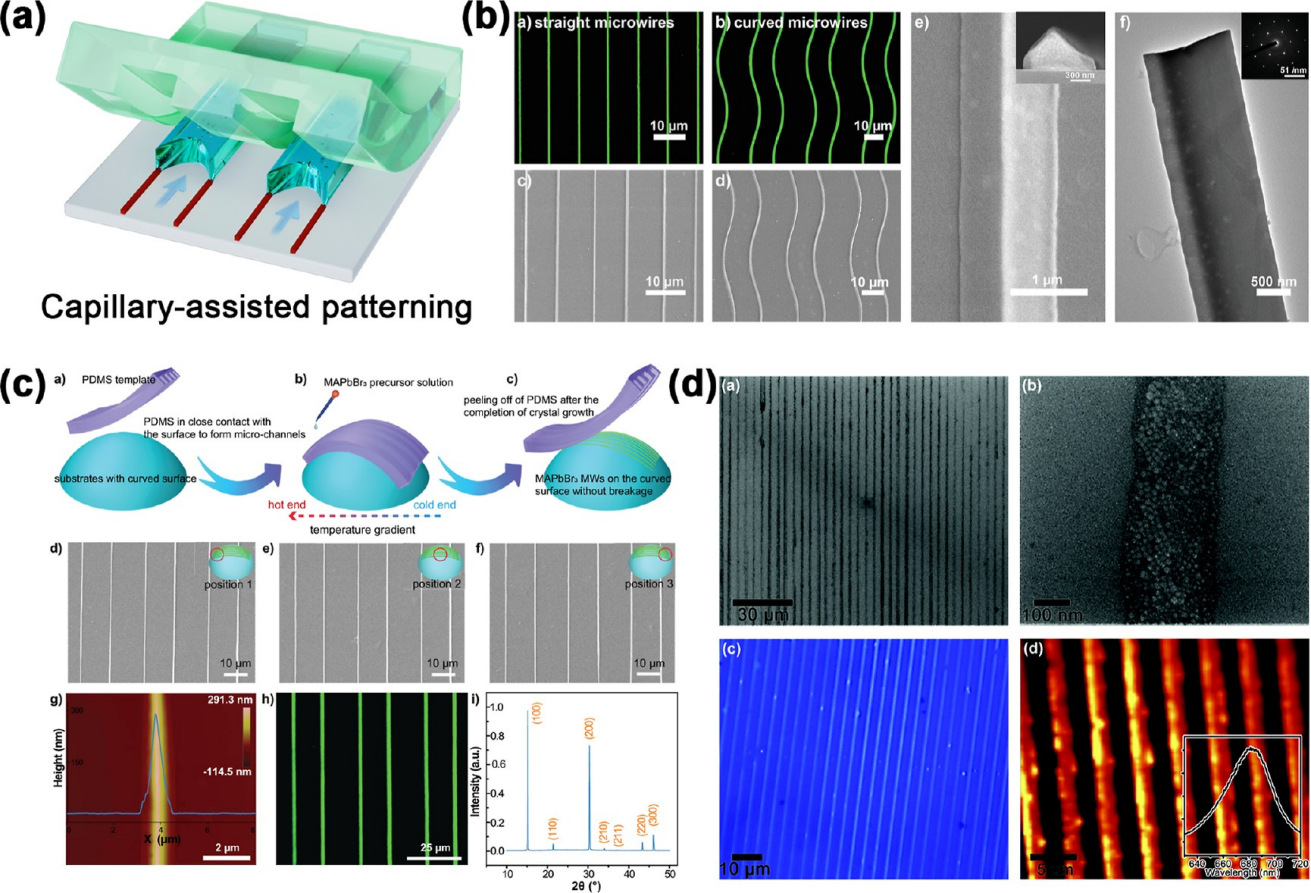
(a) Schematic diagram of capillary-assisted perovskite patterning. (b) Microscopic characterizations of MAPbBr_3_ single-crystal microwire arrays. (Reproduced with permission.^215^ Copyright 2020, Wiley-VCH.) (c) SEM, atomic force microscopy, and fluorescence microscope images of MAPbBr_3_ microwire crystal arrays on the curved surface. (Reproduced with permission.^214^ Copyright 2022, Wiley-VCH.) (d) The SEM image, large-area optical image, and PL mapping of the perovskite 1D QDs aligned by the capillary-bridge-mediated assembly on the SiO_2_/Si substrate. (Reproduced with permission.^217^ Copyright 2019, Royal Society of Chemistry.)

Li et al. bonded a PDMS template featuring periodic microgroove-protrusion patterns onto substrates, incorporating a spacer-generated venting port at the template termini for solvent vapor release. Through capillary-driven transport, the MAPbBr_3_ precursor infiltrated these microchannels, with solution adhesion to channel sidewalls forming dual liquid tails. Progressive solvent evaporation induced TCL migration along sidewalls, yielding high-purity, single-crystal MAPbBr_3_ microwire arrays. Figure 7b illustrates hydrophobic FOTS molecular transfer from PDMS to crystal surfaces, generating an in situ protective coating against water/oxygen permeation.^215^ This PDMS soft template could be pressed on a hemispherical curved substrate for in situ fabrication of curved MAPbBr_3_ perovskite microwire (MW) arrays on a curved surface. After the perovskite precursor solution dropped to the hot end of the template, MAPbBr_3_ flowed forward, driven by the temperature gradient and capillary force, and rapidly filled the microchannels. Figure 7c shows that the crystals formed ordered bent microwires along the interface of the curved substrate and the PDMS sidewalls, which prevented defects and damage caused by subsequent bending.^214^ The temperature gradient growth strategy can also be used to fabricate MAPbBr_3_–MAPbI_3_ microwire lateral heterojunctions. First, MAPbI_3_ microfilament crystals were grown at one end of the channels and maintained at 100 °C. The MAPbBr_3_ precursor was added to the other end and kept at 25 °C to prevent the initially crystallized MAPbI_3_ microfilament crystals from being dissolved by the solvent and subsequently flowing into the channels. Moreover, the system was tilted to promote and accelerate the flow of the solution in the channels, so that the force of gravity could effectively assist the flow of the solution. A high-quality MAPbBr_3_–MAPbI_3_ microwire heterojunction array was formed.^220^ Hu et al. addressed nanoscale capillary wetting limitations through capillary condensation-driven filling. Precursor immersion in sealed quartz crucibles with controlled thermal evaporation accelerated the nanocapillary infiltration. Precise hot plate temperature/duration regulation enabled condensation rate and gas molecular flux control, permitting tunable MAPbI_3_ nanowire array growth.^284^

Researchers have used liquid bridges generated by topographic templates for directed assembly of nanoparticles dispersed in solution.^285–291^ Dai et al. sandwiched perovskite quantum dots dispersed in an organic solvent between a substrate coated with a metal electrode and a silicon template with a microgroove structure, as demonstrated in Figure 7d. The organic solvent evaporated, which caused the liquid film to rupture, resulting in a regular “liquid tail” at the precise position between the sidewall of the microgroove and the supporting substrate. After further dehumidification treatment, perovskite quantum dots aggregated along the solid–liquid–gas TCL and finally formed an aligned 1D array of CsPbX_3_ perovskite quantum dots on the supporting substrate.^217^ Lee et al. naturally evaporated a quantum dot solution in the gap between an upper convex lens and a lower flat silica/silicon substrate. The controllable gap distance between the two surfaces enabled the formation of a capillary bridge for the perovskite quantum dot (QD) solution. During evaporation at rest, the evaporation loss of the solvent was greatest at the capillary edge. When the inward capillary force exceeded the immobilization force, the previously immobilized liquid rapidly moved to a new position, and the solute was replenished from the internal solution to restore the initial contact angle. The repetition of this stick–slip motion fixed/unfixed the TCL of the QD solution, and the CsPbX_3_ perovskite QDs formed a highly ordered multiple-concentric-ring pattern on the substrate during evaporation.^292^

#### 4.2.2 Wettability-Driven Template-Assisted Growth

The boundary conditions that dominate microfluidic flow can be changed by controlling the wettability of the microchannel wall.^79^ Wu’s team developed a wettability-assisted perovskite growth strategy and successfully patterned perovskites^212,213,221–224,293^ and organic crystals.^294–297^ A schematic diagram of this method for preparing perovskites is shown in Figure 8a. Feng et al. reported the preparation of CH_3_NH_3_PbX_3_ perovskite microsheet arrays via a “liquid knife” strategy implemented with wettability-mediated silicon templates with micropillar structures. A silicon substrate with a spin-coated SU-8 layer was pressed onto a topographic template with a periodic micropillar structure to selectively cover the tops of the micropillars with a thin layer of SU-8 photoresist. Then, low-surface-energy heptadecafluorodecyltrimethoxysilane (FAS) molecules were introduced onto the sidewalls, and a gap was created for hydrophobic modification. After the SU-8 protective layer was removed, a periodic micropillar arrangement with lyophobic sidewalls and a lyophilic top was obtained. The perovskite precursor liquid was immobilized on the top of the lyophilic columns, and the liquid meniscus formed a “liquid knife” that divided the liquid film, resulting in tiny domains anchored on the micropillars to limit the nucleation of the perovskite. As demonstrated in Figure 8b,c, a high-quality, uniform-sized, and precisely positioned single-crystal perovskite square microplate array was obtained.^223^ When the topographical template with asymmetric wettability described above was used, the perovskite precursor solution rose in the gap between the top of the micropillar and the flat base, driven by the capillary force and Laplace pressure, accompanied by rapid evaporation and crystallization. 2D perovskite nanowire arrays were efficiently generated on target substrates.^293^ In contrast to previous processes, another study constructed an assembly system with a sandwich configuration by bringing the PDMS stamp covered with low-surface-energy FAS molecules into contact with the top of the micropillar structure template. The perovskite precursor was added dropwise to the micropillar structure template, and the SiO_2_/Si substrate was covered. Owing to the liquid repellency at the top of the micropillars, the organic liquid was confined in the gaps between the micropillars, resulting in strictly aligned square capillaries. As the solvent evaporated, capillary tailings appeared at the lyophobic–lyophilic boundary, and a 1D single-crystal CsPbBr_3_ array grown along the [100] direction was controllably generated.^212^

**Figure 8:**
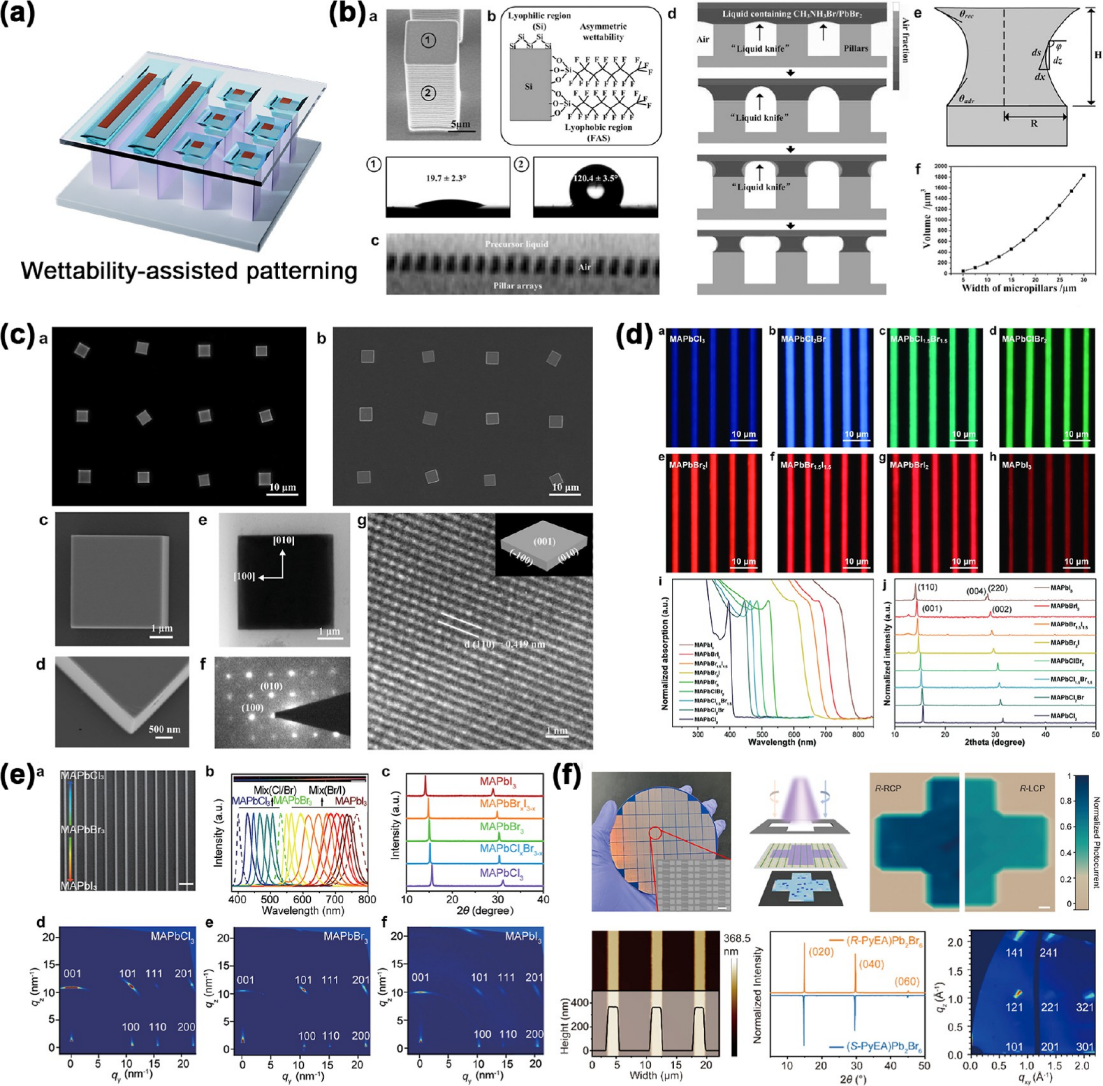
(a) Schematic diagram of wettability-assisted perovskite patterning. (b,c) Controllable dewetting and crystal growth process by the “liquid knife”. (Reproduced with permission.^223^ Copyright 2016, Wiley-VCH.) (d) Microscopic, spectral, and XRD characterization of 1D MAPbX_3_ single-crystal arrays with continuously tunable bandgaps. (Reproduced with permission.^213^ Copyright 2018, Wiley-VCH.) (e) Characterization of high-quality gradient perovskite microwire arrays. (Reproduced with permission.^221^ Copyright 2023, Wiley-VCH.) (f) Morphology and optical properties of chiral 3D perovskite microwire arrays. (Reproduced with permission.^222^ Copyright 2024, American Chemical Society.)

Gao et al. used a sandwich-type assembly system to control the capillary flow of the perovskite precursor solution between an asymmetric wettability micropillar template and a flat substrate to achieve nucleation and the formation of 1D single-crystal MAPbX_3_ perovskite arrays. Owing to the lyophilicity of the micropillars, the system was filled with solutions. As the solvent evaporated, the surface tension caused a concave meniscus to appear between the micropillars and then shrink. Driven by the Laplace pressure difference, the liquid aggregated directionally at the base and the micropillar top gap, forming a capillary bridge. As demonstrated in Figure 8d, the liquid film was split through its dewetting in the horizontal direction, and the perovskite grew into a 1D array with constrained nucleation in the gap.^213^ Zhao et al. successfully prepared (101)-oriented (ThMA)_2_(MA)_n–1_Pb_n_I_3n+1_ perovskite nanowire arrays by combining solvent engineering and capillary bridge lithography. The lyophilicity at the top of the micropillars and the lyophobicity of the sidewalls repelled the liquid from the gap of the micropillars, and the liquid was immobilized on the highly adhesive top surface of the micropillars, resulting in discrete capillary bridges after the liquid film broke up and was bound to the surface of the micropillars. Single-crystal nanowires of uniform size grew in the capillary bridges along the retracting direction of the liquid.^224^ Fu et al. used the assembly method of asymmetric wettability topographic templates combined with the micropulling technique for composition engineering, as shown in Figure 8e. The MAPbBr_3_ microwire array used as the starting material was slowly pulled out of oleylamine halide (OAmX, X = Cl, I) solution for gradient anion exchange, forming microwire arrays from MAPbCl_3_ to MAPbI_3_.^221^ Bai et al. manipulated the fluid transport dynamics through a one-step capillary bridge assembly technique and, for the first time, constructed a chiral (R/S)-1-(pyridine-4-yl)­ethan-1-amine (R/S-PyEA) Pb_2_Br_6_ perovskite-integrated device. R/S-PyEA was introduced into the precursor solution as a chiral cation. An asymmetric wettability topographic template was used to confine the liquid between the tops of the micropillars and a Au electrode substrate to form a sandwich-type assembly system. As depicted in Figure 8f, the specific nucleation and directed growth ensured the formation of strictly aligned, uniformly sized, and precisely positioned chiral perovskite microwire arrays on the target gold electrode substrate.^222^

#### 4.2.3 External Force-Driven Template-Assisted Growth

The blades not only guide the distribution and flow of a solution in a specific direction but also restrict the growth direction of crystals, as shown in Figure 9a. In particular, this method is critical for the formation of ordered single-crystal perovskite arrays.^298^ Jie’s team developed a blade-assisted and SU-8 channel-confined patterning technique to achieve large-area directional growth of perovskite microwire arrays.^210,211,216,299^ Deng et al. coated a perovskite precursor solution on a substrate heated to 100 °C and placed a spatula at the front edge of the perovskite precursor solution to form a horizontal TCL. As the solvent evaporated, CH_3_NH_3_PbI_3_ molecules precipitated at the contact line and formed nuclei, and these nuclei aggregated along the contact line. The distribution of the solution was then controlled by a spatula so that the solution was evenly spread on the substrate. CH_3_NH_3_PbI_3_ molecules continuously flowed to the growth sites and self-organized into neatly arranged single-crystal microwires via strong intermolecular interactions.^299^ To make the preparation process of perovskite crystals more controllable, SU-8 with periodic grooves was used as a microreactor, and the precursor solution was evenly coated and filled with a scraper blade, precisely defining the perovskite precursor. The flow and growth space of the bulk solution were set to achieve precise control over the growth of the perovskite crystals. As demonstrated in Figure 9b, the microchannels not only helped stabilize the transport of perovskite solutes but also reduced the density of nucleation events, ensuring the formation of a uniform and continuous single-crystal array in the channels.^211^ Sun et al. proposed a 3D constrained crystallization strategy to prepare centimeter-sized single-crystal organic–inorganic hybrid perovskite arrays with a high crystalline quality. The researchers chose a PDMS triangular prism mold as the spatula for solution shearing. Because the movement speed of the spatula was close to the crystallization speed of MAPbI_3_, the exposed perovskite solution rapidly evaporated and crystallized at the front end of the spatula when heated at 145 °C. As the PDMS spatula continued to move, the perovskite precursor solution continued to be delivered to the crystallization site to replenish the depleted solute; thus, the perovskite crystals continued to grow, and eventually, a large-area MAPbI_3_ crystal array was formed in each microchannel. The PDMS mold not only fit closely to the surface of the microchannels to form a closed 3D microsized space but also avoided damage to the microchannels during shearing of the solution and effectively prevented solution adhesion,^216^ as illustrated in Figure 9c.

**Figure 9:**
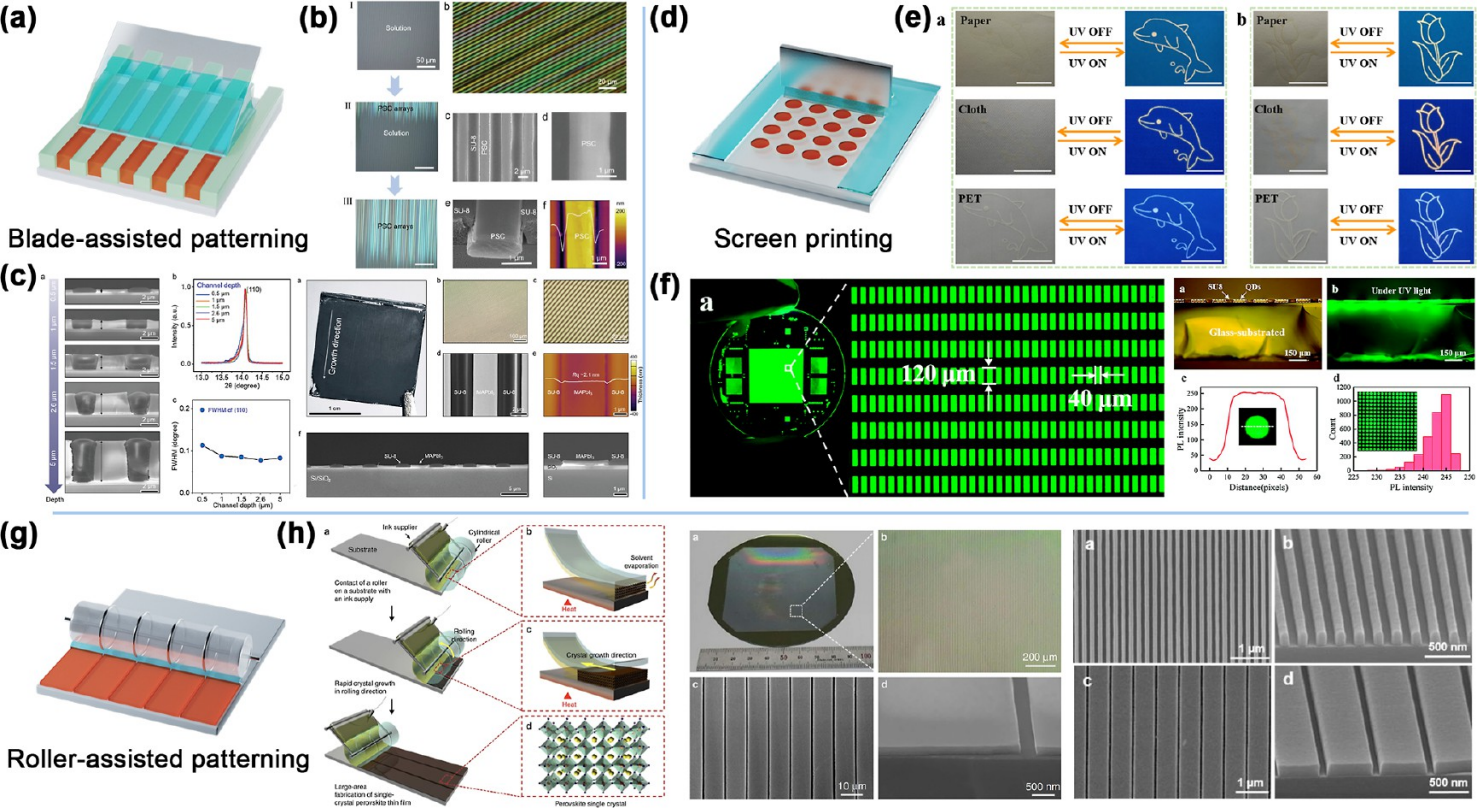
(a) Schematic diagram of blade-assisted perovskite patterning. (b) Cross-polarized optical microscopy image of the CH_3_NH_3_PbI_3_ PSC arrays at a 45° rotation angle with respect to the axis of the crossed polarizers. SEM and AFM images of the CH_3_NH_3_PbI_3_ PSC arrays. (Reproduced with permission.^211^ Copyright 2020, Wiley-VCH.) (c) Cross-sectional SEM images of the MAPbI_3_ crystals grown from microchannels with different depths at a fixed width. Characterization of the MAPbI_3_ single-crystal array. (Reproduced with permission.^216^ Copyright 2022, Wiley-VCH.) (d) Schematic diagram of patterned perovskite fabrication by screen printing. (e) Encryption and decryption of the information by the tertiary-color luminescent perovskite inks printed on paper, cloth, and PET. (Reproduced with permission.^300^ Copyright 2021, American Chemical Society.) (f) Optical and fluorescence images of the perovskite quantum dot color conversion layer. (Reproduced with permission.^301^ Copyright 2022, Royal Society of Chemistry.) (g) Schematic diagram of roller-assisted perovskite patterning. (h) Microscopic characterization of the perovskite thin films fabricated on a wafer-scale Si substrate by the geometrically confined lateral crystal growth process. (Reproduced with permission.^302^ Copyright 2017, The Authors, published by Springer Nature.)

Sequential deposition of different perovskite layers is usually technically challenging when wet chemical methods are used because the solution of the latter perovskite layer can dissolve the previously deposited perovskite layer.^303^ Xie et al. demonstrated a PDMS-template-assisted sequential printing method to fabricate MAPbBr_3_–MAPbI_3_ perovskite heterostructure arrays. First, a flexible PDMS template with periodic grooves was tightly attached to a substrate and a MAPbBr_3_ solution was injected to form aligned liquid columns. Solvent evaporation led to the formation of crystal nuclei at the tips of the liquid column array, which grew directionally along the microchannels to form a 1D MAPbBr_3_ array. Subsequently, via a blade coating process, a PDMS template with rectangular cavities was filled with MAPbI_3_ precursor solution and then inverted on the preprinted MAPbBr_3_ array, in which the long-range orientation of the rectangular cavities was strictly aligned with that of the MAPbBr_3_ array. The MAPbI_3_ solution could be controllably and uniformly crystallized at specific locations of the MAPbBr_3_ array to form a MAPbBr_3_–MAPbI_3_ heterostructure array.^218^

Screen-printing coats the substrate simply by rapidly sweeping the scraper on a patterned metal or polyester screen loaded with screen-printing paste.^304,305^ A schematic diagram of screen printing for preparing patterned perovskites is shown in Figure 9d. The core of this technology relies on a high-viscosity perovskite ink. Such an ink not only exhibits enhanced cohesion and adhesion to the substrate but also meets the demand for a high-viscosity ink in screen printing and can be used to precisely manufacture nanoscale films in 3D space and achieve full contact between the ink, substrate, and pattern.^306,307^ Chen et al. developed an aqueous luminescent ink based on MAPbBr_3_@PbBr­(OH) NCs, which were synthesized through a grinding process in the presence of 2-methylimidazole (2-MIM) and oleylamine (OAm). A water-based perovskite ink suitable for screen printing was prepared by adjusting the formulation and adding an alkali-soluble acrylic resin, a defoamer, a thickener, etc., to increase the viscosity of the ink. The ink was printed on a substrate via a screen-printing plate. As shown in Figure 9e, the ink printed on the substrate through the screen printing plate forms an encrypted information pattern after film curing, which can be decrypted under UV light irradiation.^300^ Sun et al. reported the fabrication of high-resolution patterned MAPbBr_3_ QD–polymer composite arrays with a pixel size of 2–100 μm via the SU-8 template-based micropore filling method. First, SU-8 microwell plates were prepared via photolithography. A perovskite QD gel (a powder made of the precursor and PMMA mixed with PDMS) was subsequently dropped into the SU-8 microporous mold. Finally, the gel was filled into the micropores via a spatula and solidified at 70 °C to form QD pixels,^301^ as demonstrated in Figure 9f.

Roll-to-roll printing and slot-die coating have advantages in the fabrication of large-scale perovskite devices.^308–310^ Figure 9g is a schematic diagram of roller-assisted perovskite patterning. Lee et al. wrapped a flexible PDMS mold with an array of channels 10 μm wide and 200 nm deep with a 400 nm-wide spacing on a cylindrical metal roller. The rolling die was then placed in contact with a preheated SiO_2_ substrate, and the filled perovskite ink solution immediately crystallized at the open ends of the channels in the vertical direction. The deposited ink solution was vertically confined between the substrate and channels of the mold. As shown in Figure 9h, the crystal growth in the vertical direction was limited, inducing lateral growth of CH_3_NH_3_PbI_3_ crystals.^302^

### 4.3 Inkjet Printing Technology

Inkjet printing is becoming an emerging trend in the manufacture of crystalline optoelectronic devices because of its maskless, noncontact, and material-efficient characteristics.^311^ By finely regulating the ink ejection frequency, ink droplet volume, and print speed of the printhead, this technology can print arrays of different sizes and densities, as illustrated in Figure 10a. The printability of an ink formulation is quantified by the Ohnesorge number (Oh), which takes into account the rheological properties of the ink, such as the density (ρ), shear viscosity (η), and surface tension (σ), and the given nozzle diameter (d).^312^ These parameters are usually expressed in the form of dimensionless Reynolds numbers (*Re* = vρd/η) and Weber numbers (*We* = v^2^ρd/σ), which are derived from the Navier–Stokes flow equation.

$$Oh=\frac{1}{Z}=\frac{\sqrt{We}}{Re}=\frac{\eta }{\sqrt{\rho \sigma d}}$$

**Figure 10:**
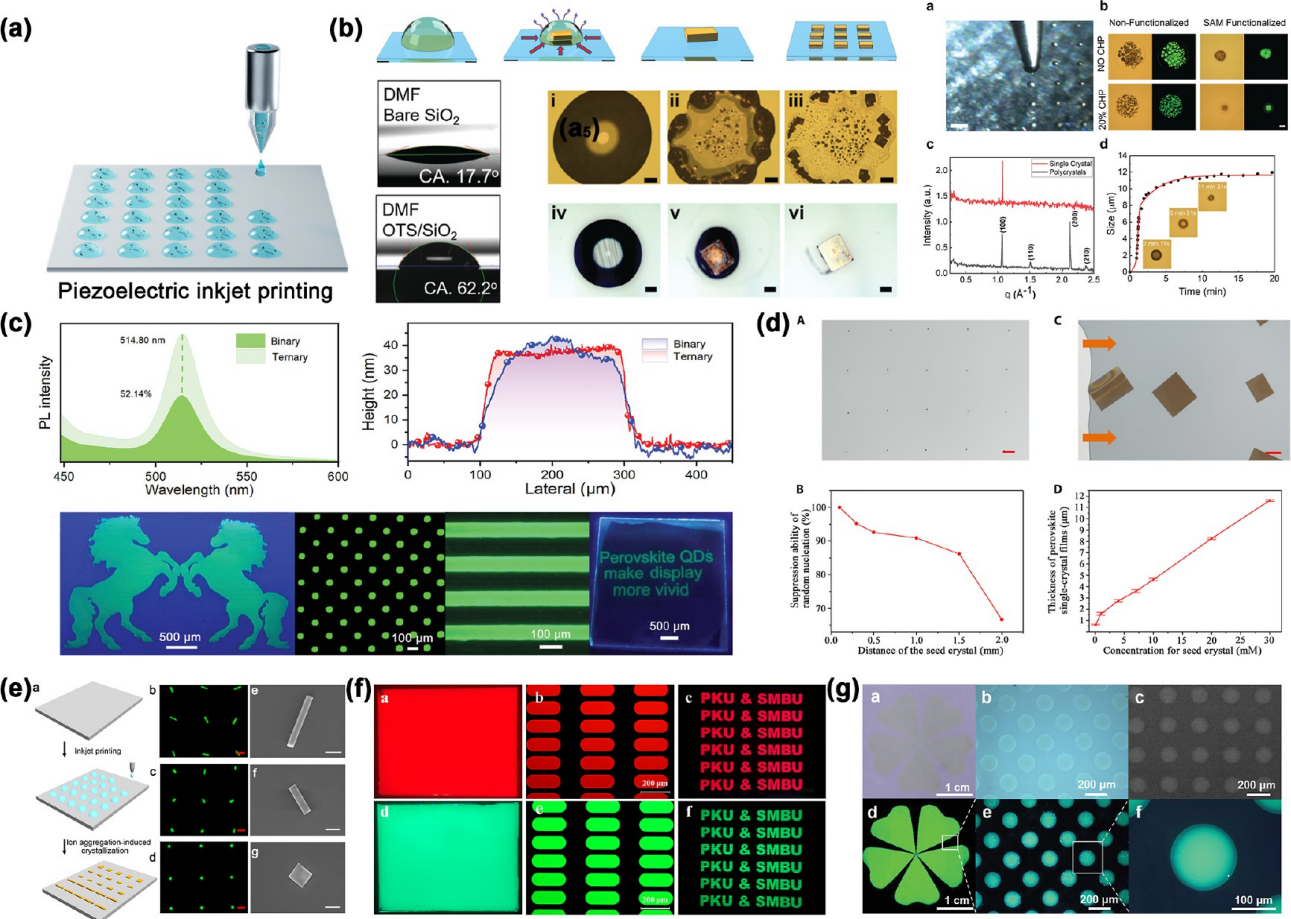
(a) Schematic diagram of perovskite array fabrication using piezoelectric inkjet printing. (b) Images showing the contact angle and the drying process at different stages of a droplet on a bare SiO_2_ substrate and an OTS-functionalized substrate. Screenshot and microscopy images during the printing of MAPbBr_3_ crystals. (Reproduced with permission.^166^ Copyright 2020, Wiley-VCH.) (c) PL spectra, topographical profiles, and fluorescence optical microscopy images of inkjet-printed QD thin films. (Reproduced with permission.^160^ Copyright 2022, Wiley-VCH.) (d) Optical images of patterned perovskite seeds and their growth process, the relationship between random nucleation suppression ability and seed distance, and the dependence of perovskite single-crystal film thickness on seed size. (Reproduced with permission.^157^ Copyright 2018, The American Association for the Advancement of Science.) (e) Photoluminescence and SEM images of the CsPbBr_3_ single-crystal arrays with different morphologies. (Reproduced with permission.^147^ Copyright 2023, American Chemical Society.) (f) Photoconversion performance of red and green CsPbX_3_ NC patterns. (Reproduced with permission.^193^ Copyright 2024, Wiley-VCH.) (g) Optical and microscopy images of patterned perovskite films under light field, dark field, and UV light. (Reproduced with permission.^314^ Copyright 2019, Wiley-VCH.)

The *Z* value in the range 4–14 is expected to indicate good printability. At a low *Z* value, the separation of droplets is hindered, whereas at a high value, the formation of many satellite droplets is more likely to occur.^313^ In general, solvents with high boiling points and low VPs are beneficial for preventing nozzle clogging and premature crystallization of the precursor ink.

#### 4.3.1 Piezoelectric Inkjet Printing

##### 4.3.1.1 Direct Printing

Under the action of a piezoelectric pulse, a piezoelectric material is squeezed, bent, pushed, and sheared, which causes deformation of the ink chamber wall, thus forcing the ink to be ejected from the nozzle. There are no strict requirements for volatile components or temperature stability. In contrast, thermal inkjet technology requires the formation of air bubbles, which are limited to vaporizable inks and are not suitable for organic solvent-based inks. Therefore, the latest methods for inkjet printing optoelectronics almost completely rely on piezoelectric printing.^315^ The challenge in direct printing of perovskite arrays lies in inducing fast and uniform nucleation while slowing down crystal growth to obtain a thermodynamically favorable orientation and larger grains.^316^ Wang et al. introduced phenylbutylammonium bromide (PBABr) to generate a narrow phase distribution in the perovskite, which could reduce nonradiative recombination and improve the photoluminescence quantum yield (PLQY) of perovskite thin films. Then, a vacuum-assisted rapid drying process was used to achieve high-quality quasi-2D CsPbBr_3_ perovskite films without the coffee ring effect.^154^

The perovskite crystallite morphology also strongly depends on the combined effect of the evaporation rate and the wettability of the substrate.^144,150^ For example, researchers have used a PVP layer to improve the wettability of substrates and suppress the coffee ring effect by increasing the Marangoni flow strength to spread droplets and form a uniform perovskite thin film on a substrate.^185,188^ Alternatively, plasma can be used to irradiate a substrate to improve the adhesion of ink droplets to the surface, and a perovskite film with small pinholes and large grains can be deposited by optimizing the substrate evaporation temperature.^140^ Corzo et al. proposed a cosolvent evaporation strategy. The introduction of CHP, which has a high boiling point, a high surface tension, and a low solubility, as a cosolvent into printing inks allows main solvents such as DMF, DMSO, or GBL to evaporate. The solvent evaporates first, resulting in a supersaturated solution. Rapid crystal growth consumes the solute and prevents additional nucleation, ensuring that each droplet generates a single crystal. OTS hydrophobic treatment and a high-surface-tension cosolvent together effectively regulate the wettability, unpinning the TCL such that the droplets shrink inward during evaporation to inhibit the formation of the coffee ring effect. The method, as shown in Figure 10b, minimizes parasitic crystallization events on the substrate, enabling the formation of single-crystal MAPbBr_3_ perovskite arrays.^166^

##### 4.3.1.2 Quantum Dot Printing

Perovskite quantum dots (QDs) refer to nanocubes with sizes in the strong quantum-confinement regime, where their optical properties are predominantly governed by their size.^317^ Inkjet printing has emerged as the dominant technique for patterning perovskite QDs.^318^ This is primarily because QD inks, as dispersions of solid nanoparticles, circumvent the core issue of nozzle clogging due to the crystallization of perovskite precursor inks. Additionally, the composition, size, and optical properties of QDs are fixed during ink synthesis, meaning that no significant chemical changes occur after printing, thereby ensuring consistent performance before and after patterning.

Ionic perovskite QDs are highly sensitive to polar solvents because lattice distortion and phase transition of perovskite QDs may be triggered by polar molecules through van der Waals attraction. Therefore, in solvent engineering of perovskite QD inks, attention should be given to the dispersibility, orthogonality, and printability of the system.^319^ Wei et al. proposed a universal ternary solvent ink strategy utilizing cycloalkane, *n*-tridecane, and *n*-nonane to produce the CsPbX_3_ perovskite QD ink with high dispersibility and stability. Figure 10c shows the resulting ink with better printing suitability and film-forming ability than traditional inks.^160^ Zhang et al. added the surfactant l-α-phosphatidylcholine (LP) to a CsPbBr_3_ QD solution presynthesized through thermal injection and encapsulated the CsPbBr_3_ QDs in silica to prepare CsPbBr_3_/LP/SiO_2_ QD composites with a high PLQY and good color purity. Owing to the synergetic effect of the surfactants and core/shell structures in improving the dispersion stability and controlling the growth kinetics, the stability of the QDs toward water, ambient oxygen, and UV light was fully improved.^199^

##### 4.3.1.3 Seed-Crystal Printing

By combining inkjet printing with solution processing methods, single-crystal perovskite arrays with various compositions and morphologies can be fabricated on a large scale using prenucleated seed crystals.^141,147,157,158^ Gu et al. used a perovskite precursor solution as an ink and successfully prepared size-controllable CH_3_NH_3_PbX_3_ multicolor single-crystal microplatelets by adjusting the inkjet volume of the ink as well as the effects of the adhesion force of the substrate and the evaporation temperature on perovskite crystallization.^141^ This seed crystal array could be used to grow millimeter-sized single-crystal perovskite films with controlled thickness and high throughput on a large scale, and the film thickness could be flexibly adjusted over a large range through the seed size. The seed crystal arrays prepared via inkjet printing were placed on substrates covered with a saturated perovskite precursor solution. Under drying at room temperature, the seed crystals effectively inhibited random nucleation and promoted in situ growth of a single-crystal film on the seed crystals by affecting the mass transfer and changing the distribution of the perovskite precursor ions,^157^ as shown in Figure 10d. The anisotropic growth of perovskite single crystals can be further controlled by adjusting the evaporation temperature to achieve the selective printing of perovskites with controllable morphologies and growth positions. Figure 10e displays diverse morphologies, such as single-crystal CsPbBr_3_ perovskite microwires, microstrips, and microplate arrays.^147^ This seed growth strategy can also be applied in vapor phase growth to selectively epitaxially grow single-crystal CsPbBr_3_ microplate arrays with uniform morphology and controlled positions and size, overcoming the lattice mismatch and random nucleation barrier.^158^

##### 4.3.1.4 Polymer Encapsulation Printing

Owing to the limitations of vertical printing of droplets, perovskite materials are easily exposed to the external environment, and their poor stability and crystal brittleness affect their practical application. The coffee ring effect and miscibility problems generated during inkjet printing often led to poor crystallization and uneven thickness of the films, reducing device performance. Achieving a balance between outward capillary flow and inward Marangoni flow is key to eliminating the coffee ring effect.^144^ The polymer–perovskite composite ink printing method can relieve the coffee ring effect generated during evaporation, and the perovskite can be in situ crystallized inside the polymer to isolate it from the external environment. An ideal polymer should have the same solvent solubility as the perovskite precursor to ensure effective phase mixing and dielectric properties compatible with the device.^208^

Researchers have added PVP to perovskite inks to regulate the viscosity of the perovskite precursor and thus control the internal flow resistance and external evaporation rate of the perovskite ink and eliminate the capillary flow that causes the coffee ring effect during evaporation. Under space limitations of PVP, CsPbBr_3_ PeNC–PVP composite microarrays with uniform size distributions have been fabricated in situ.^184,186,187^ Shi et al. demonstrated in situ thermal curing preparation of MAPbBr_3_ QDs/PVA by inkjet printing using water as a solvent. At higher temperatures, the QDs and polymer can solidify within a similar time and induce crystallization of MAPbBr_3_ QDs embedded in PVA, eventually forming a patterned thin-film array.^190^ Additionally, in situ encapsulation and patterning of perovskite QDs were achieved by mixing a UV-curable acrylic resin, a photoinitiator, and a perovskite QD solution as a special ink suitable for inkjet printing. The addition of the polymer significantly increased the contact angle of the droplets and slowed their evaporation. Figure 10f shows that the good dispersion of perovskite QDs in the polymer matrix facilitated the formation of thin films with a uniform surface morphology distribution.^193^ Polymerization of this ink was activated under UV radiation, further generating a tightly cross-linked polymer network, which effectively encapsulated the perovskite QDs to protect them from environmental influences, and the ink cured into a regular three-dimensional microarray pattern.^192–195^

The use of PeNCs embedded in polymer matrices is an in situ encapsulation method based on the polymer swelling effect.^49,320–324^ This scheme can avoid the use of polymer-containing inks in the printing process and has a wide range of applicability to various perovskites and polymers.^325^ For example, Shi and Jia et al. demonstrated in situ inkjet printing strategies for MAPbX_3_ QDs and quasi-2D PeNC patterns, respectively. As shown in Figure 10g, the perovskite precursor ink is inkjet-printed onto a polymer film on a heated substrate for dissolution or swelling and crystallization into QDs within the polymer matrix.^314^ Since DMF or DMSO is a common solvent in ink, this strategy can be used to fabricate perovskite QD patterns on different polymer films, such as PMMA, PS, polycarbonate (PC), polyvinyl chloride (PVC), polyvinylidene fluoride (PVDF), polyvinylidene chloride (PVDC), cellulose acetate (CA), and polyacrylonitrile (PAN).^314,326^ Gu et al. used liquid-to-liquid self-encapsulation inkjet printing technology to directly print a perovskite ink into a liquid PDMS precursor to form a PDMS structure in situ with single-crystal MAPbBr_3_ perovskite embedding. The steric confinement effect of the liquid PDMS precursor could significantly delay the perovskite crystallization process and promote intercalation growth of single crystals in PDMS. Owing to the sealing function of PDMS, the printed perovskite single crystal exhibited excellent environmental stability and flexibility.^327^

#### 4.3.2 Electrohydrodynamic Printing

Excessively reducing the nozzle diameter to improve the printing resolution becomes impractical since extremely high pressure will be required to overcome the capillary force. However, pulling the liquid from a nozzle tip by applying an electric field is relatively easy. Therefore, electrohydrodynamic (EHD) printing technology can jet liquid droplets with smaller diameters to the designated positions on the substrate, breaking the resolution bottleneck of traditional inkjet technology.^328–330^ The electric field causes the mobile ions in the ink to gather in a region near the surface of the pendant meniscus. The Coulomb repulsion between these ions deforms the meniscus into a conical shape, which is named the Taylor cone. When a high voltage potential is applied, a droplet is ejected from the cone when the electrostatic stress overcomes the surface tension, as shown in Figure 11a. This method is capable of direct patterning of materials with a resolution extending down to the submicroscale.^191,331^

**Figure 11:**
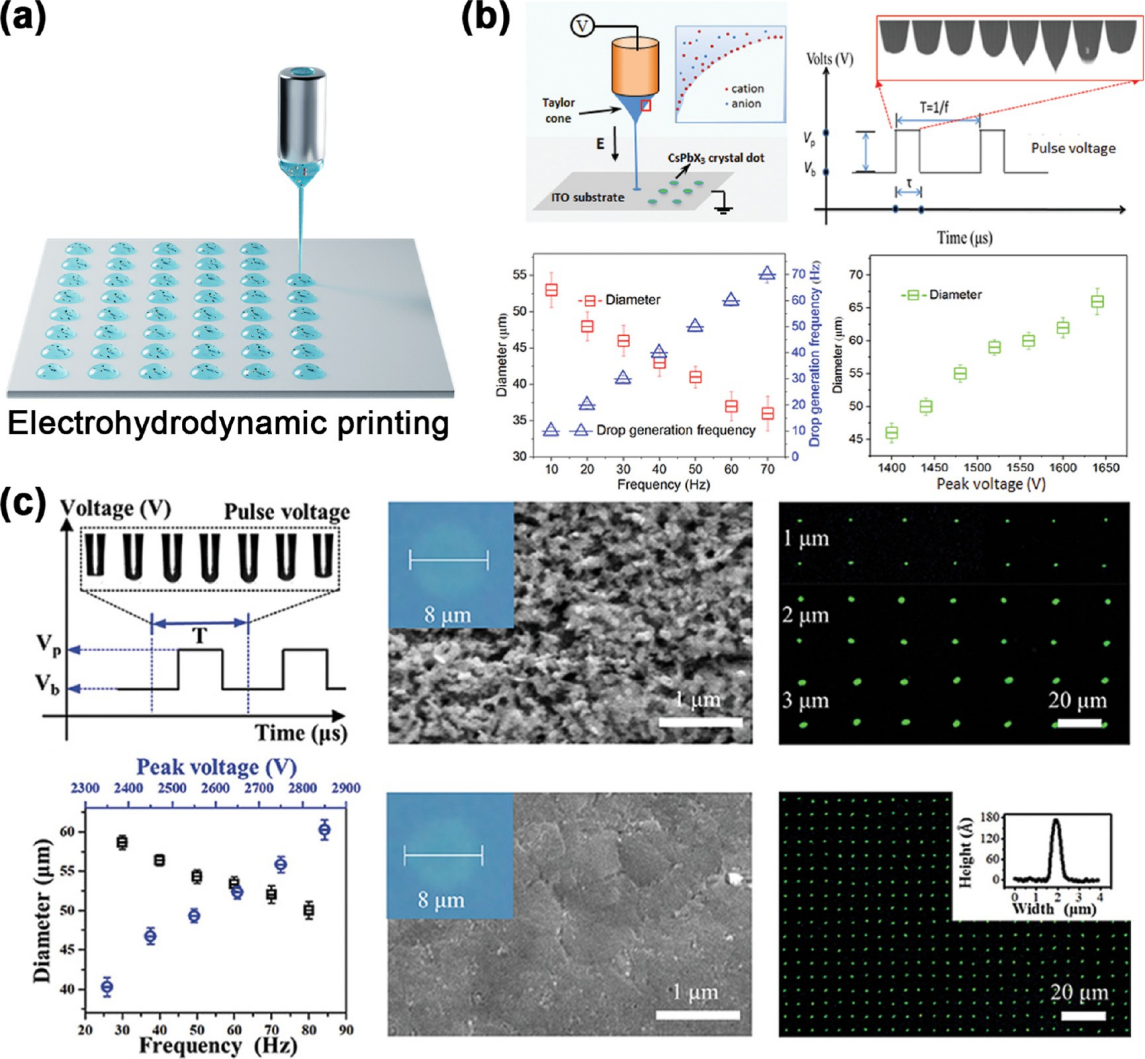
(a) Schematic diagram of perovskite array fabrication using EHD printing. (b) Schematic of the experimental setup for the EHD printing system. CsPbX_3_ crystal dot diameter as a function of jetting frequency, pulse voltage frequency, and voltage pulse peak. (Reproduced with permission.^332^ Copyright 2019, Wiley-VCH.) (c) MAPbX_3_ dot diameter versus pulse frequency and peak voltage. An image of the EHD-printed high-resolution dot array. (Reproduced with permission.^189^ Copyright 2021, Wiley-VCH.)

Zhu et al., with the help of 2-phenylethanamine bromide (PEABr) and 18-crown-6 additives, realized a 5 μm high-resolution full-color CsPbX_3_ dot matrix through the EHD printing process, as demonstrated in Figure 11b. PEABr plays an important role in the morphology of the film, whereas 18-crown-6 helps control the phase separation and the distribution of the crystallite size.^332^ Wang et al. used ionic liquid methyl acetate (MAAc) as a solvent to improve film quality by reducing the perovskite growth rate. EHD printing of full-color MAPbX_3_ perovskite dot arrays with a resolution of down to 1 μm was achieved. Figure 11c shows that MAAc overcomes the issues associated with traditional microdroplets, such as the use of DMF and DMSO as solvents, which are prone to producing more pinholes and crystal defects because of excessive evaporation and insufficient flow and crystallization time.^189^ Yang et al. introduced a dual-ligand passivation strategy to stabilize PeNCs and inhibit the migration of halogen ions during the high-pressure EHD printing process. Lecithin was used as the main ligand to reduce structural damage to the CsPbBrI_2_ NCs under the electric field. Dodecanethiol (1-DT) was used as an auxiliary ligand to passivate the halogen vacancies on the surface of the PeNCs. A perovskite array with a minimum pixel size of 5 μm was realized.^178^

#### 4.3.3 3D Printing

Inkjet-printing-based patterning technologies are still limited to in-plane manufacturing and alignment, whereas 3D printing technology allows the free production of three-dimensional structures in space, which can expand the application of modern optoelectronics in terms of free circuits and high integration density requirements. Chen et al. developed a 3D printing technology based on a programmed thermal drawing process for nanopipettes, in which the femtoliter meniscus of the precursor ink was used to induce evaporation and crystallization of the perovskite to produce 3D perovskite nanostructure arrays with a preferred crystal alignment.^225–227^ Figure 12a shows a schematic diagram of the 3D printing fabrication of perovskite arrays. The perovskite crystallization process was driven by the rapid evaporation of the DMF solvent at the meniscus of the precursor ink in the pipet. By stretching the ink to perform wiredrawing, the diameter and hollowness of CH_3_NH_3_PbI_3_ nanostructures could be controlled on demand,^225^ as illustrated in Figure 12b. Other precursors, such as CH_3_NH_3_PbBr_3_ and CH_3_NH_3_PbCl_3_ perovskites, can also be used to guide the highly confined, out-of-plane crystallization process via the meniscus, thereby achieving crystallization to fabricate 3D perovskite nanopixels with programmed size, position, and red, green, and blue (RGB) emission characteristics,^226^ as shown in Figure 12c. By using a dual-tube nanopipette with a height difference as the printing nozzle, the stepped geometry design allows sequential double printing. Figure 12d shows that the 3D fabrication of a CH_3_NH_3_PbX_3_ heterostructure can be realized in a few seconds.^227^

**Figure 12:**
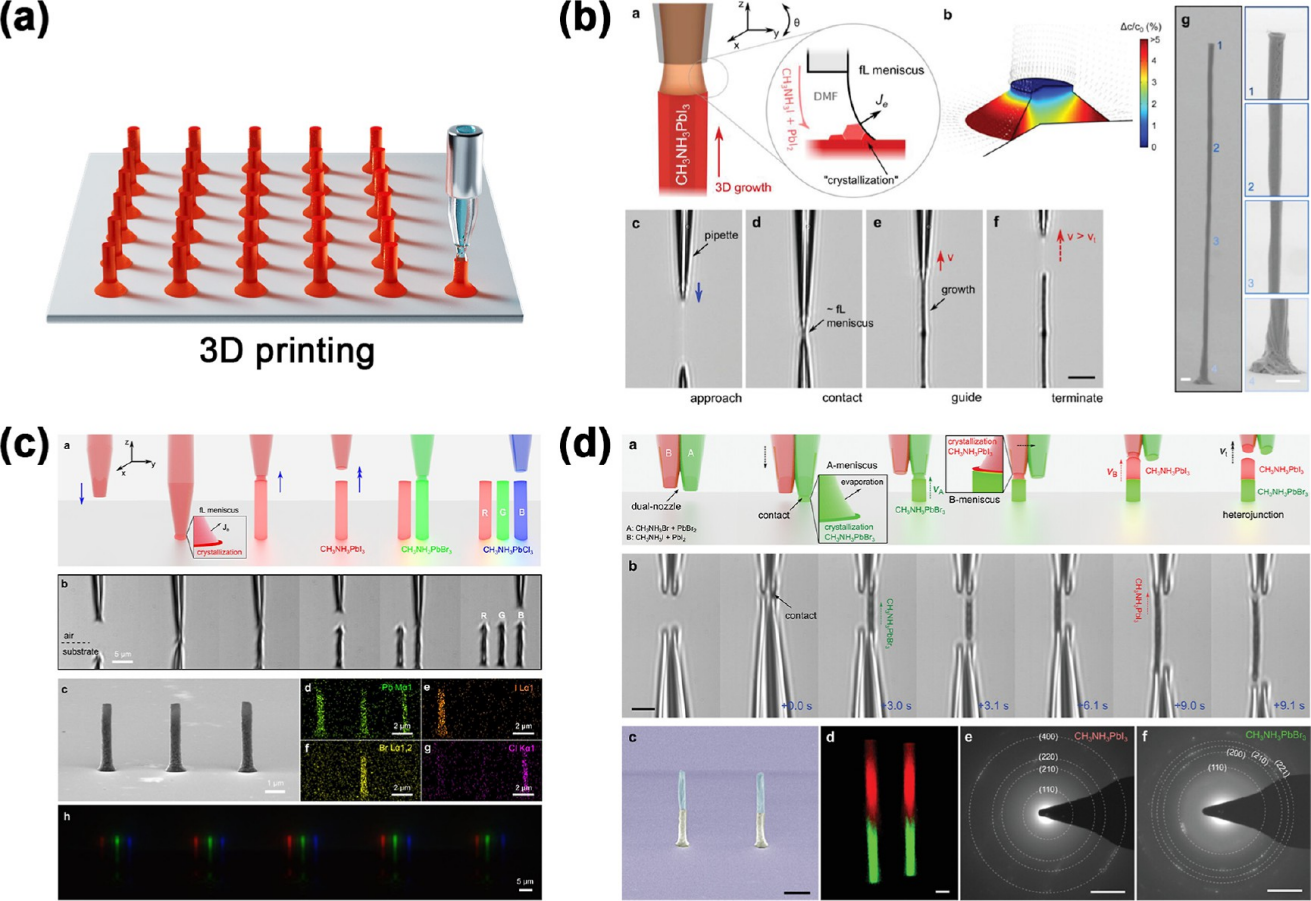
(a) Schematic diagram of perovskite array fabrication using 3D printing. (b) Schematic illustration showing meniscus-guided 3D printing of organic–inorganic metal halide perovskites. Optical microscopy images of the 3D printing process. (Reproduced with permission.^225^ Copyright 2019, Wiley-VCH.) (c) SEM and energy-dispersive X-ray spectroscopy and optical photoluminescence images of an as-printed perovskite RGB triple pixel consisting of CH_3_NH_3_PbX_3_ nanopillars. (Reproduced with permission.^226^ Copyright 2021, American Chemical Society.) (d) False-colored SEM image of perovskite nanowire heterojunctions fabricated by 3D printing. (Reproduced with permission.^227^ Copyright 2023, Wiley-VCH.)

Peng et al. used dual-nozzle inkjet printing to prepare single-crystal perovskite patterns and 3D structures by mixing the perovskite precursor with an antisolvent. In the first step, the precursor solution was inkjet-printed, and then the prepared antisolvent solution was printed at the edge of the immobilized precursor droplets. The edge deposition strategy was used to minimize the contact area between droplets, which could confine the crystal nucleation sites within a minimal coalescence area. The inkjet printing parameters were adjusted to set the crystal growth direction, and precursor/antisolvent printing cycles were continuously performed at a fixed position to create cylindrical 3D crystal structures with heights of up to mm.^167^ Chen et al. added the polymer PS and a nonpolar xylene solvent to the PeNC colloidal ink to fabricate full-color CsPbX_3_ PeNC arrays with 3D micropillar structures on rigid and flexible substrates. The hydrophobic PS polymer could not only shield PeNCs from environmental water vapor but also prevent ions from migrating across grain boundaries under an electric field disturbance, effectively protecting the lattice structure of PeNCs and maintaining their photoelectric stability. The morphology of the 3D perovskite was regulated by changing the pulse voltage and pulse duration to achieve a minimum diameter of 2.8 μm and a maximum height of 24 μm.^198^

### 4.4 Contact Printing Technology

In a contact printing process, physical contact occurs between a printing plate and a substrate and an ink is patterned on or transferred from the printing plate to the substrate through the application of pressure. Perovskite thin films with complex structures and high-density arrays can be prepared by precisely customizing the printed patterns.

#### 4.4.1 Nanoimprinting

Nanoimprint technology uses a prepatterned stamp to impress a liquid on a substrate, as depicted in Figure 13a. The physical sidewalls or protrusions of the template restrict the flow of the liquid on the substrate, ensuring a uniform distribution and stability of the droplets at the intended positions. Unlike the semiconfined template-assisted method, this technique does not require capillary tubes to introduce the liquid. Therefore, it is suitable for solvents that do not spontaneously wet the substrate or the mold.^333^ Park’s team developed an imprint technique for patterning perovskite films with different compositions.^334–336^ For example, Jeong et al. used a prepatterned PDMS mold to imprint a perovskite precursor liquid film in the soft gel state. Figure 13b shows that MAPbBr_3_ perovskite micropattern arrays were obtained.^334^ The compressibility of the precursor solution was improved by adding a small amount of poly­(ethylene oxide) (PEO) to further increase the resolution of the patterning of CsPbX_3_ perovskite to 200 nm, including periodic lines, squares, hexagonal holes, and rectangles,^335^ as demonstrated in Figure 13c. Park et al. developed a nanoimprint combined with a block copolymer-guided self-assembly technique for large-area fabrication of 1D nanopatterns of various 2D perovskites with a scale of sub-30 nm (A′_2_MA_*n*–1_Pb_*n*_X_3*n*+1_, A′ = BA, PEA, X = Br, I). This technology uses a hard PDMS mold to achieve highly ordered pattern transfer through replication of guided self-assembled block copolymer nanopatterns. A small amount of poly­(2-vinylpyridine) (P2VP, a strong Lewis-base polymer additive) was subsequently added to the perovskite precursor solution to limit perovskite crystallization and provide sufficient time for the subsequent imprinting process. Then, the DMSO solvent was evaporated via heat treatment, and 1D perovskite nanopatterns with high crystallinity over large areas were obtained.^336^

**Figure 13:**
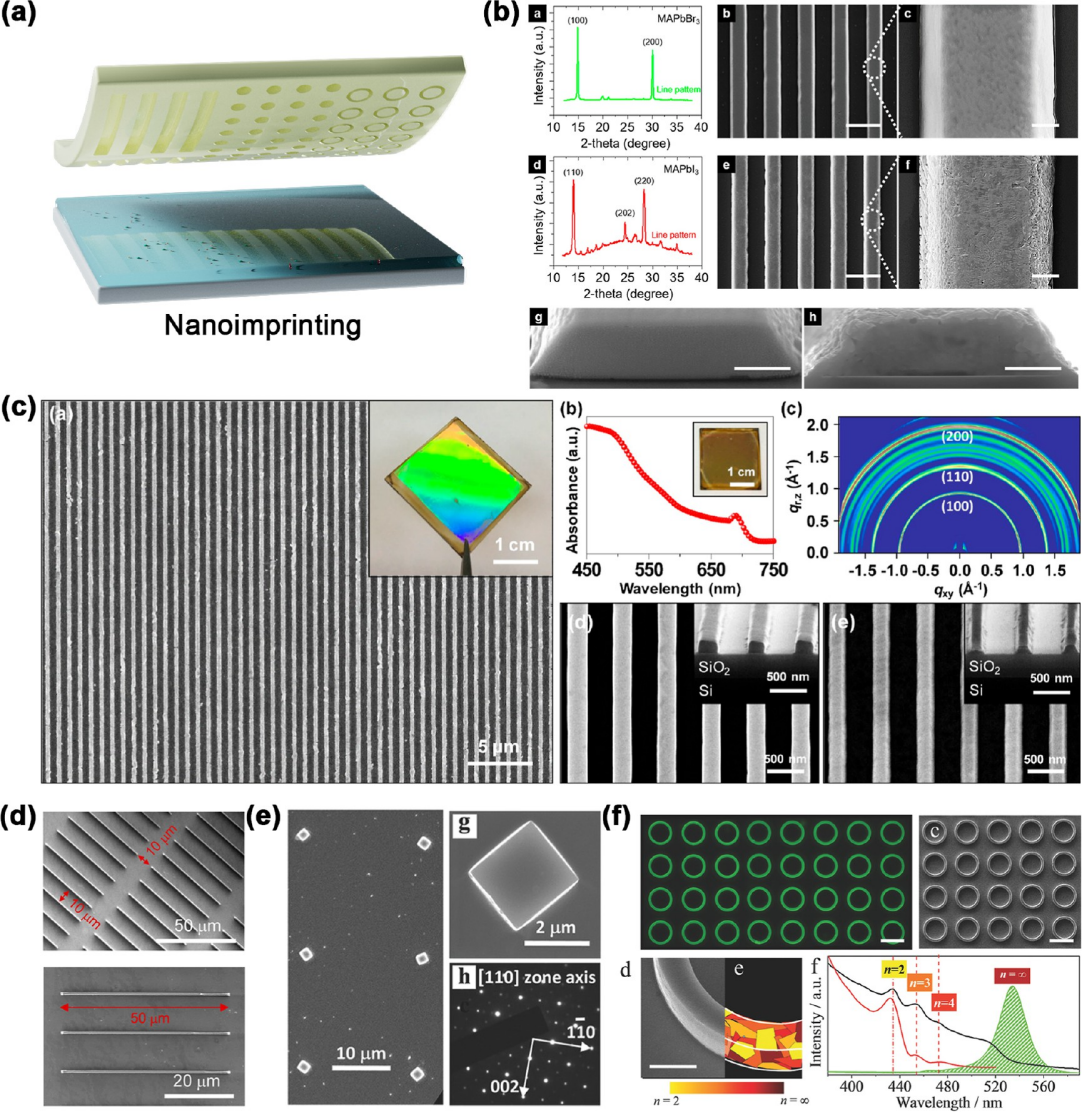
(a) Schematic diagram of nanoimprinting fabrication for patterned perovskites. (b) XRD patterns and SEM images of periodic lines of MAPbX_3_ film prepared by solvent-assisted gel printing. (Reproduced with permission.^334^ Copyright 2016, American Chemical Society.) (c) SEM images, absorbance spectrum, and grazing incidence wide-angle X-ray scattering pattern of the perovskite nanopattern processed by polymer-assisted nanoimprinting with a polymer additive. (Reproduced with permission.^335^ Copyright 2020, American Chemical Society.) (d) SEM images of MAPbBr_3_ perovskite nanowire arrays. (Reproduced with permission.^337^ Copyright 2017, American Chemical Society.) (e) In situ monitoring of the PDMS cylindrical-hole-template confined solution growth of CsPbCl_3_ microdisks. SEM images, XRD spectra, and PL spectra of CsPbX_3_ rectangular microdisks. (Reproduced with permission.^233^ Copyright 2017, Wiley-VCH.) (f) Fluorescence images, SEM images, and PL spectra of perovskite microring arrays with strict alignment and precise positioning. (Reproduced with permission.^338^ Copyright 2018, Wiley-VCH.)

Fu’s team prepared perovskite arrays with various morphologies and sizes by using a PDMS template with specific pore shapes combined with solution self-assembly.^233,337,338^ Liu et al. lifted the PDMS cast on a patterned silicon wafer to form a groove template with an array arrangement. This template was then pressed onto a SiO_2_/Si substrate, and the perovskite precursor was filled into the gaps to form a spatially controlled linear droplet array. As presented in Figure 13d, with the slow evaporation of DMF, the perovskite nucleated at the end of the template and underwent directed growth along the channel, forming a size-controllable (width of 460–2500 nm; height of 80–1000 nm; length of 10–50 μm) MAPbX_3_ perovskite nanowire array.^337^ He et al. used a cylindrical PDMS pore template to contact a perovskite precursor solution to an OTS-pretreated hydrophobic SiO_2_/Si substrate and applied mild pressure to drive the solution into the pores. The slow evaporation of DMF induced the growth of the perovskite under steric confinement into a single-crystal rectangular microdisk array. Figure 13e shows that CsPbX_3_ perovskite arrays with a side length of 2.5 ± 0.3 μm, a thickness of 0.6 ± 0.2 μm, and a controllable spacing were realized on any substrate.^233^ Zhang et al. further prepared large-area microring arrays of 2D organic–inorganic hybrid Ruddlesden–Popper perovskites by using a concave microring-shaped PDMS template,^338^ as demonstrated in Figure 13f. This method has strong versatility and can be applied to a variety of perovskite materials to form different morphologies, such as nanowires, microsheets, and microrings, flexibly meeting application needs.

Apart from the commonly used silicon templates,^339^ optical discs are inexpensive and readily available materials whose surfaces contain microscale or nanoscale structural features, which greatly reduces the cost of custom templates. For example, Lu et al. used PDMS to replicate the 1D nanogratings of commercial CD-ROMs and DVD-ROMs as pattern templates and achieved crystallographically aligned MAPbI_3_ perovskite nanowire arrays with variable line widths and alignment densities via imprinting.^340^ Wang et al. also used a CD as a PDMS template to imprint a PVP-stabilized CsPbI_3_ film onto a nanowire array. Then, a layer of PDMS gel was scrape-coated on the nanowires, and half of it was peeled off after curing. CsPbI_3_–CsPbBr_3_ lateral heterogeneous nanowire arrays were prepared via gas–phase ion exchange.^219^

The nanoimprint technique is also suitable for fabricating perovskite films with grating structures.^341^ A mold with good thermal stability and pressure resistance is placed on a perovskite precursor soft gel under a certain pressure, and the periodic pattern is copied from the mold to the perovskite layer. Finally, the high-crystallinity microstructure of the perovskite film is completed by annealing and demolding.^342–347^ A diffraction grating with a continuous perovskite microstructure was constructed, which achieved nanoscale photon capture, enhanced light extraction and charge transport through diffraction, and effectively inhibited carrier recombination.^348^ The highly crystalline perovskite film and nanograting structure endowed photodetectors with excellent polarization characteristics.^55,349–352^

#### 4.4.2 Transfer Printing

In transfer printing, nanomaterials or a paste are transferred to a target substrate via a stamp often made of PDMS, which has a low surface energy and is an ideal viscoelastic stamp material.^353–357^ In this method, by optimizing the printing pressure, temperature, and surface wettability, perovskite deposition with high resolution and high uniformity is achieved, as illustrated in Figure 14a. This method avoids the defects and heterogeneity caused by solvent evaporation during direct deposition. To prevent internal cracking of PeNC films during transfer printing, Li et al. spin-coated a perovskite film directly onto a PDMS substrate with a concave pattern. The PDMS substrate with the perovskite layer was then pressed onto a silicon wafer with a concave pattern and was slowly picked up. Then, the CsPb­(Br_0.84_Cl_0.16_)_3_ perovskite pattern on the PDMS substrate was printed on the target substrate. This method can achieve a transfer rate of approximately 100%.^358^ Kwon et al. developed a dual-layer transfer printing technique for perovskite and organic charge transport layers to overcome the cracking problem of CsPbX_3_ thin films. After spin-coating a perovskite layer on a donor substrate, a 2,2′,2″-(1,3,5-benzinetriyl)-tris­(1-phenyl-1-*H*-benzimidazole) (TPBi) layer was thermally evaporated. As shown in Figure 14b, a PDMS stamp was then used to transfer the pattern onto the receiving substrate, and perovskite patterns with RGB subpixels were created after repeated pad printing.^359^ Zhou et al. fabricated a colorful concentric circle pattern array of MAPbBr_3_ PeNCs by pressing a PDMS-hexane solution-coated hard Si pillar stencil onto a flat substrate coated with the precursor solution. In this process, the interference of the light reflected at the top and bottom interfaces of the droplet generated a Newton ring phenomenon, which could be used to evaluate the height of the droplet. Perovskite single crystals with different morphologies were prepared by adjusting the column depth, spin-coating speed, and solution viscosity.^360^

**Figure 14:**
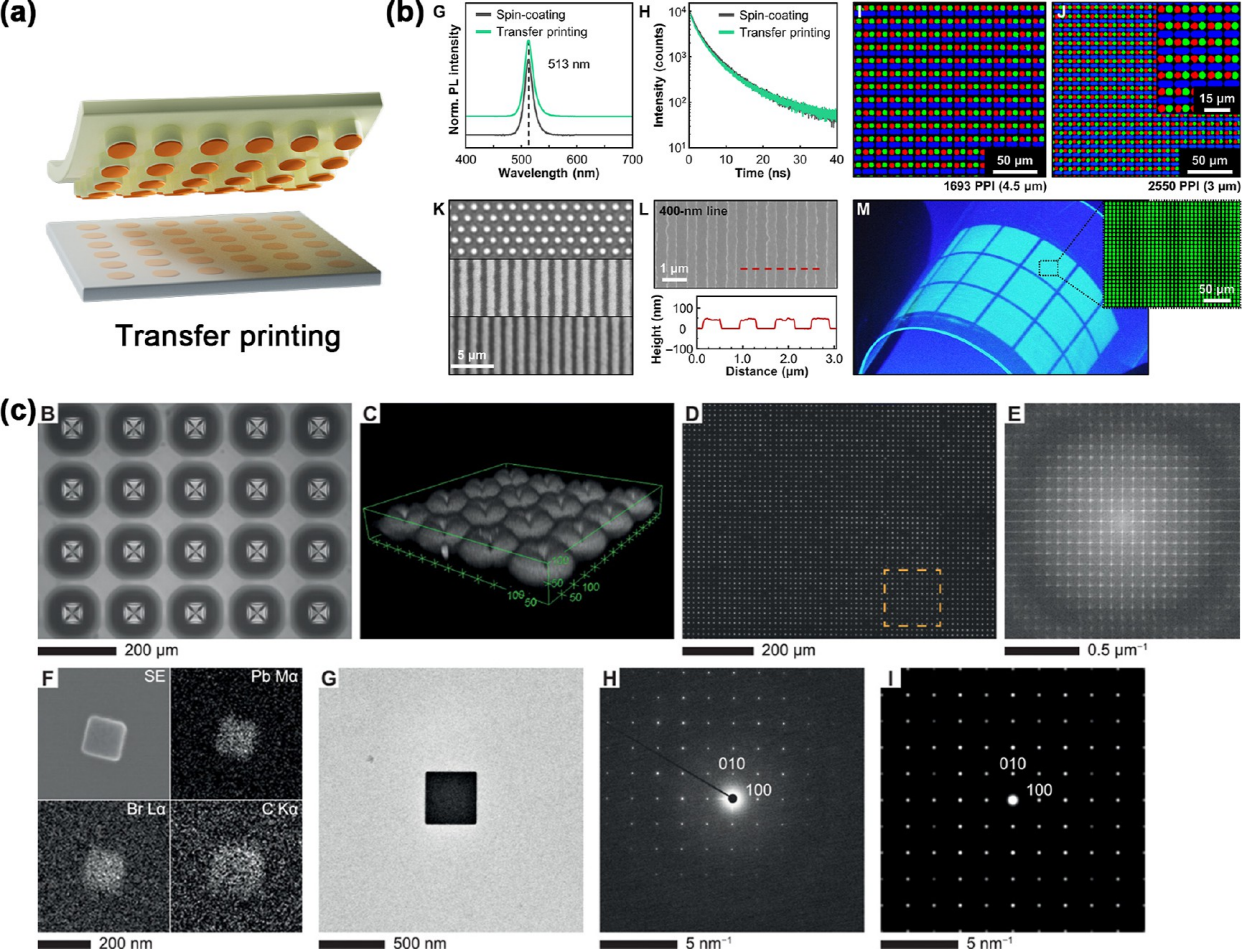
(a) Schematic diagram of transfer printing for preparing patterned perovskites. (b) Fluorescence microscopic images, SEM images, and AFM topography results of the pixelated RGB perovskite nanocrystal patterns. (Reproduced with permission.^359^ Copyright 2022, The American Association for the Advancement of Science.) (c) Schematic illustration of the synthesis process for the halide perovskite nanocrystal arrays. SEM images of perovskite nanocrystals with various extension lengths. (Reproduced with permission.^361^ Copyright 2020, The American Association for the Advancement of Science.)

Du et al. proposed a method combining polymer pen lithography and contact printing to prepare MAPbBr_3_ PeNC arrays. First, the perovskite ink was spin-coated onto an array of approximately 1000 PDMS pyramidal pens. As illustrated in Figure 14c, owing to the high surface tension and low viscosity of the ink, the ink aggregated around the base of each pyramid and acted as a continuous ink reservoir. A soft elastomer tip was used to deliver the ink to the substrate surface via direct writing. Owing to the high surface area-to-volume ratio, the nanoreactors readily vaporized within seconds, resulting in nucleation and growth of individual halide PeNCs. Since each PDMS pyramidal pen created 121 crystals, more than 100,000 droplet nanoreactors could be deposited on the substrate with high throughput. This nanoreactor-based synthesis method satisfies the feature size control requirements of nanolithography and provides a large-area manufacturing ability.^361,362^

### 4.5 Microfluidic Droplet Generation Technology

Microfluidic technology uses microchannels to process immiscible liquid phases to deform and pinch off the target liquid to generate discrete microdroplets surrounded by another liquid in a high-throughput manner.^363,364^ Compared with other patterning platforms, the microfluidic platform performs high-precision dynamic synthesis of material particles in a closed environment, avoids external contamination, and enables real-time in situ characterization.^365^ Figure 15a shows a schematic diagram of the perovskite array fabricated by using a microchannel-based microfluidic platform. Li et al. injected a CsPbX_3_ QD solution into microchannels and transported the solution to 140 × 50 μm pixels. As shown in Figure 15b, the solid QDs formed by solvent volatilization acted as a color-conversion array, and there was space between the blue LEDs and the QDs to prevent heat transfer. Finally, the outlet and inlet of the microchannels were sealed to protect the QDs from degradation in air and water vapor.^366^ Zhou et al. studied the mechanism of PNW growth via nanocrack-assisted micro/nanofluid manufacturing technology. First, a stress concentration and/or release structure was used to control the initiation and propagation of nanocracks to obtain a nanocrack channel that connected two microfluidic channels.^367–369^ A drop of MAI/PbI_2_/DMF solution was then loaded into the microchannel device to flow. As illustrated in Figure 15c, the initial nucleation sites and growth paths were simultaneously controlled by the guidance of the nanochannel. The constrained micro/nanochannel network enabled precise control of the growth of MAPbI_3_ nanowires.^370^

**Figure 15:**
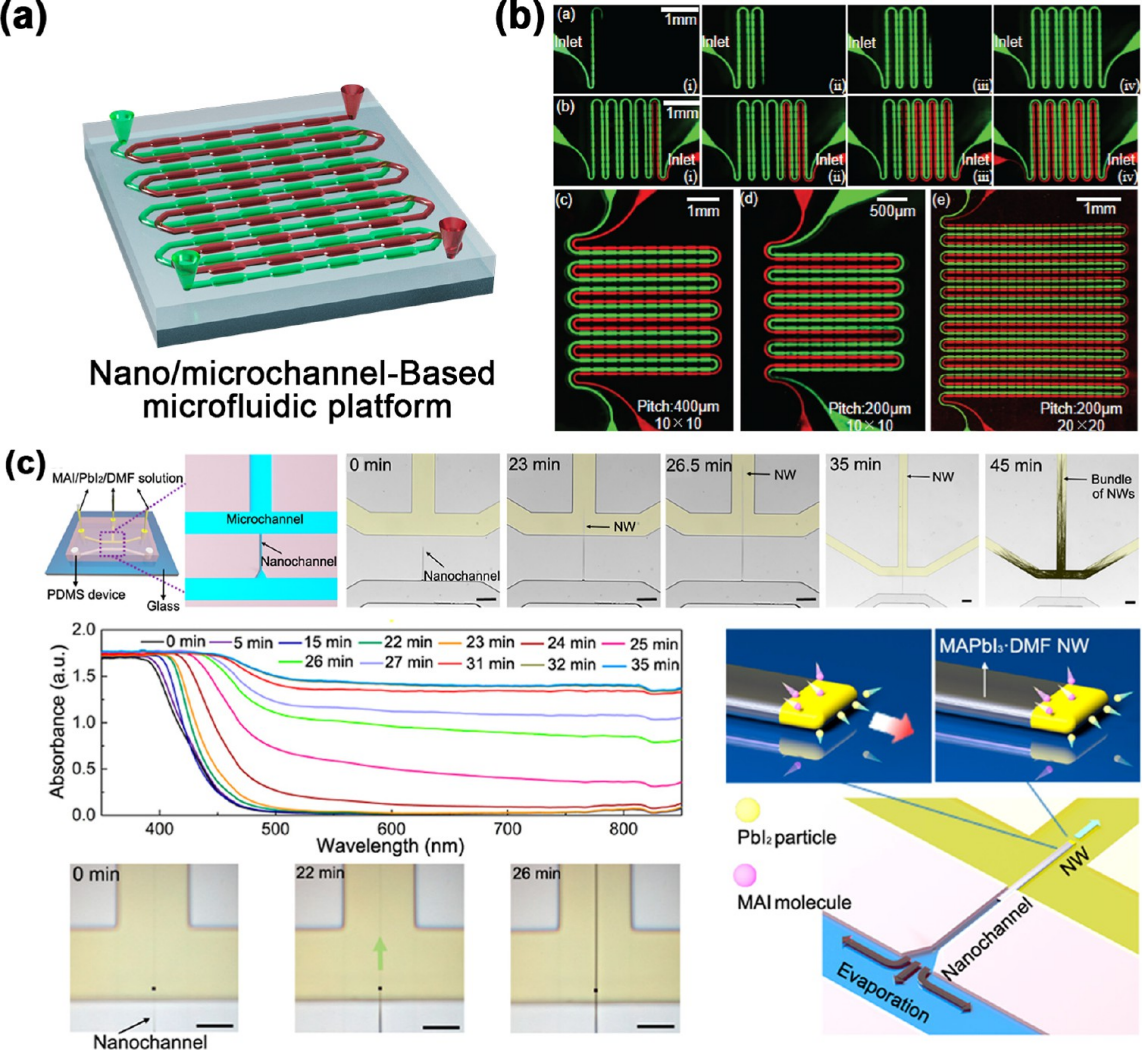
(a) Schematic diagram of perovskite array fabrication using a nano/microchannel-based microfluidic platform. (b) Optical photograph of the microchannel structure and fabrication process for the green and red perovskite quantum dot color conversion layers. (Reproduced with permission.^366^ Copyright 2021, AIP Publishing.) (c) Growth mechanism of MAPbI_3_-DMF NWs within micro/nanochannels and in situ UV–vis absorption spectra characterization. (Reproduced with permission.^370^ Copyright 2018, American Chemical Society.)

Microfluidic technology improves sample consistency by reducing the reaction volume, which eliminates product variations in the traditional manual NC synthesis due to inconsistent reaction conditions, uneven heating, or insufficient mixing.^371^ Moreover, microfluidic technology allows the creation of temperature and reagent gradients on an ultrashort time scale, and the ratio of halide ions in the perovskite can be accurately adjusted through fast and controlled mass transfer, as illustrated in Figure 16a.

**Figure 16:**
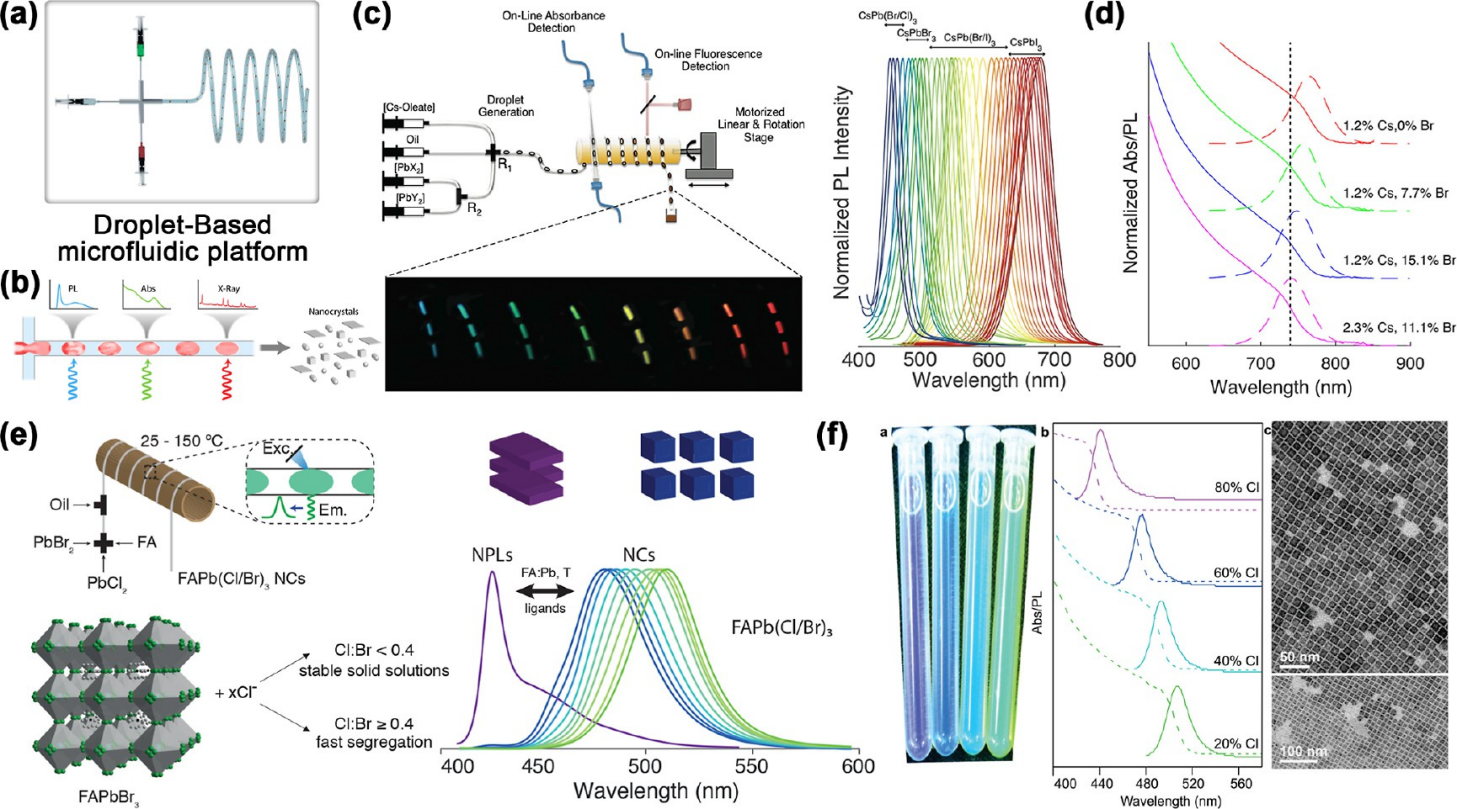
(a) Schematic diagram of perovskite array fabrication using a droplet-based microfluidic platform. (b) Schematic illustration of the droplet-based microfluidic platform for nanocrystal generation and in situ characterization. (Reproduced with permission.^372^ Copyright 2017, American Chemical Society.) (c) Image and online fluorescence spectra of the generated CsPbX_3_ nanocrystals. (Reproduced with permission.^373^ Copyright 2016, American Chemical Society.) (d) PL spectra of colloidal Cs_*x*_FA_1–*x*_Pb­(Br_1–*y*_I_*y*_)_3_ NCs synthesized using the microfluidic platform and representative online PL and online absorption spectra at different quantities of Cs^+^ and Br^–^ in the reaction mixture. (Reproduced with permission.^374^ Copyright 2018, American Chemical Society.) (e,f) A conceptualization of the interplay between the reaction parameters and the resulting shape of nanocrystals or nanoplates. Photograph and TEM images of FAPb­(Cl_1–*x*_Br_*x*_)_3_ NCs. (Reproduced with permission.^375^ Copyright 2018, American Chemical Society.)

The teams of deMello and Kovalenko proposed the use of a droplet-based microfluidic platform for the synthesis of NCs,^372^ as illustrated in Figure 16b. A reduction in the reaction volume ensures better uniformity of the thermal and chemical environments.^373–378^ Lignos et al. premixed PbX_2_ and PbY_2_ precursor solutions in a T-shaped connection mixer and then delivered them to a cross mixer, and the reaction time was controlled by controlling the residence time of the droplets in the heating zone. Figure 16c shows that the Pb/Cs molar ratio and the halide molar ratio (Br/Cl or I/Br) could be continuously and independently adjusted to generate CsPb­(X/Y)_3_ droplets with various compositions. One ″synthesis run″ required only a few mL of reagents and 1–5 h of experimental time and could generate information equivalent to that obtained in 200–1000 batches of experimental reactions.^373^ Figure 16d–f shows that the platform realized stable synthesis of pentad Cs_*x*_FA_1–*x*_Pb­(Br_1–*y*_I_*y*_)_3_ PeNCs with PL emission between 690 and 780 nm^374^ and FAPb­(Cl_1–*x*_Br_*x*_)_3_ NCs with PL emission between 440 and 520 nm,^375^ respectively. Maceiczyk et al. reported a microfluidic-based parametric screening study of FAPbBr_3_, FAPbI, and mixed halide FAPb­(Br/I)_3_ NCs. The in situ optical characterization system equipped on this platform revealed different growth mechanisms of iodide and bromide, indicating that the formation mechanism of FAPbBr_3_ NCs involved the formation of nanosheets as a transient substance, whereas FAPbI_3_ directly formed cubic NCs.^376^ Bezinge et al. reported the synthesis of (Cs/FA)­Pb­(I/Br)_3_ and (Rb/Cs/FA)­Pb­(I/Br)_3_ NCs in a high-throughput segmented flow microfluidic reactor. A self-optimizing algorithm was used to rapidly identify the reagent concentrations needed to generate user-defined PL peak wavelengths in the green–red spectral region.^377^ In summary, the microfluidic platform allows high-throughput spectroscopic measurement and reaction parameter screening. The synthesis parameters extracted from the microfluidic platform (pL-nL scale) can be completely transferred to traditional reaction flasks (mL scale) for scale-up of the production of perovskites.

## 5 Applications of Perovskite Arrays

Perovskite material arrays not only improve the consistency of their performance but also lay the foundation for the construction of high-performance photonic and optoelectronic devices. By precise control of the arrangement and spacing of the materials, arraying technology significantly optimizes the optical absorption, emission, and transmission characteristics of perovskites, significantly enhancing the optical efficiency and ensuring the stability and reliability of devices in practical applications. In this section, the important role of perovskite arrays in the entire process from simple structures to complex functional devices is emphasized, and their flexibility and high efficiency have become important driving forces for the future development of optoelectronic devices.

### 5.1 Patterned Photonic Devices

In applications such as perovskite anticounterfeiting, inkjet printing technology with low cost and high patterning accuracy has broad prospects, as illustrated in Figure 17a. By designing an arrangement of droplets to form complex anticounterfeiting patterns, a fluorescent 2D code and a barcode consisting of thousands of perovskite film dot pixels have been realized.^187,199^ These patterns are invisible in the ambient environment and are compatible with flexible substrates. Researchers have further used the fluorescence characteristics of perovskite materials to construct fluorescence-lifetime-encoded tags for fast reading, endowing them with additional covert security features,^328^ as demonstrated in Figure 17b. The vertical height of 3D-printed perovskite pixels can be used as an additional dimension to encode data that cannot be accessed by traditional wide-field microscopy due to its limited depth of field, thus providing multilevel anticounterfeiting security.^226^ In addition, the differential transport of perovskite precursor droplets, random crystallization of ionic crystals, and Ostwald ripening endow perovskite patterns with multilevel security characteristics.^116,156,260^ As shown in Figure 17c, these characteristics include macroscopic security patterns, microscopic unclonable textures, and fluorescence information.^116^ Another researcher used the correlation between perovskite nanorod arrays of random lengths prepared via the surface confinement method and laser modes to convert encoding rules based on the laser mode number into a quaternary cipher key array.^149^ The difference in the solubilities of different components of mixed halogen perovskites tends to cause component segregation, leading to their crystals exhibiting random multiwavelength emission characteristics in a specific wavelength range. The encoding pattern exhibited by the PL spectrum of such perovskite crystal arrays can be applied to all-photon code motifs.^259^ The optical characteristics of these random and irregular crystals make the identification of anticounterfeiting labels more difficult and avoid the common risk of cloning.

**Figure 17:**
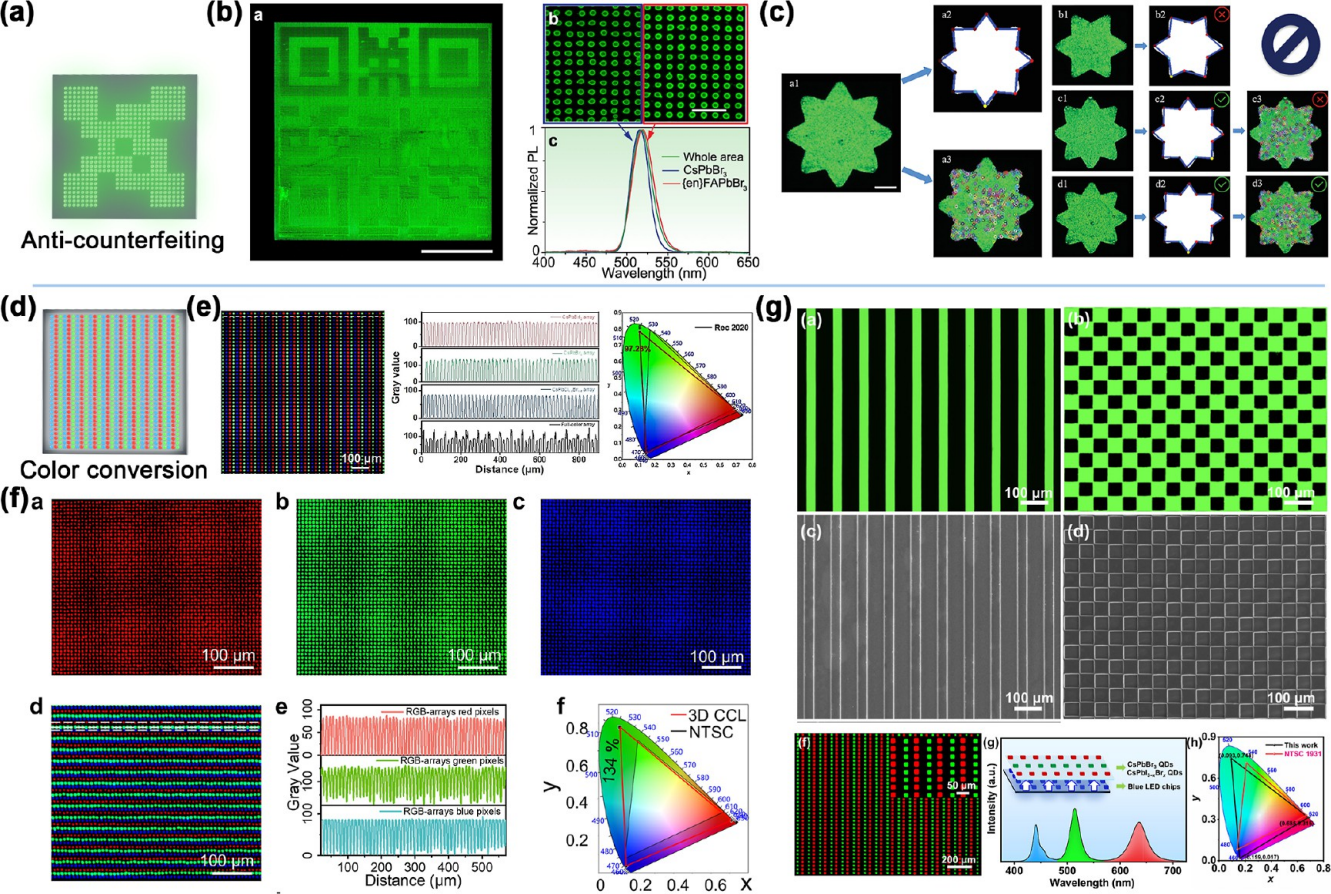
(a) Schematic diagram of the perovskite anticounterfeiting label. (b) A true-color image and PL spectra of a unicolour fluorescent QR code from PeNC inks. (Reproduced with permission.^328^ Copyright 2021, The Authors, Published by Springer Nature.) (c) The fluorescence images of perovskite film with clonable shape and unclonable texture for anticounterfeiting information. (Reproduced with permission.^116^ Copyright 2021, Wiley-VCH.) (d) Schematic diagram of the perovskite color conversion layer. (e) Array printing demonstration of tricolor PeNC arrays on a black photoresist template. The gray value and color gamut of as-printed arrays. (Reproduced with permission.^178^ Copyright 2024, American Chemical Society.) (f) Fluorescence images, fluorescence intensity distribution curves, and color gamut of the full-color 3D PeNC color conversion layer arrays. (Reproduced with permission.^198^ Copyright 2024, American Chemical Society.) (g) Fluorescent microscopy image of two-color square perovskite patterns. EL spectra, color coordinates, and gamut of the RGB hybrid perovskite quantum dot arrays. (Reproduced with permission.^191^ Copyright 2024, Springer Nature.)

In color conversion applications, developing fine color-conversion pixel patterns compatible with micro-LED or organic LED backplanes to obtain vivid display characteristics is critical.^193^ Easy-to-pattern perovskite materials have a high absorption coefficient and excellent PL quantum efficiency, with their characteristics exhibiting an extremely narrow spectral width. As illustrated in Figure 17d, the perovskite color conversion layer enables the display of highly saturated pure colors, which significantly enhances the color gamut range of displays. EHD printing drives droplets via an electric field, enabling the generation of droplets with nanometer-scale diameters, and has been used to prepare high-resolution multicolor color conversion layers,^178,198^ as demonstrated in Figure 17e,f. However, because the absorption coefficient of perovskite materials is approximately 10^5^ cm^–1^, the ideal thickness of perovskite materials used as color-conversion layers should be in the range of microns.^379^ In the inkjet printing technique, perovskite inks based on thermosetting or UV-curable additives can be used to effectively fabricate multicolor perovskite patterns with microscale thicknesses for use in color-conversion layers of full-color displays. As shown in Figure 17g, the cross-linking process ensures the uniform distribution of perovskite QDs, effectively alleviating the coffee-ring effect and improving the overall quality of the 3D microarray.^190–195^ Additionally, a microfluidic chip consisting of a glass substrate bonded to a PDMS mold with microchannels was used to precisely direct multicolor perovskite QDs to the locations of micro-LED pixels. This method operates at room temperature and does not cause damage to the QDs. A sealed system can prevent the degradation of QDs by air and water vapor.^366^

High-quality perovskite microcrystal arrays with ultralow-threshold single-mode lasing have shown great promise in lasing and can meet the ever-increasing demands for high information density and accuracy of highly integrated photonic devices, as depicted in Figure 18a. By controlling the nucleation and growth of perovskite crystals, researchers have produced single-crystal microplates,^63,148,223^ single-crystal microwires,^339^ and polycrystalline microrings^338^ with uniform sizes, regular morphologies, and high positioning accuracies for whispering gallery mode laser arrays, as shown in Figure 18b,c. Furthermore, the well-defined sizes and uniform geometry of PNWs enable single PNWs to act as high-quality Fabry–Perot nanolasers with almost the same optical mode and similar low lasing thresholds, enabling them to be simultaneously ignited as a laser array.^337^

**Figure 18:**
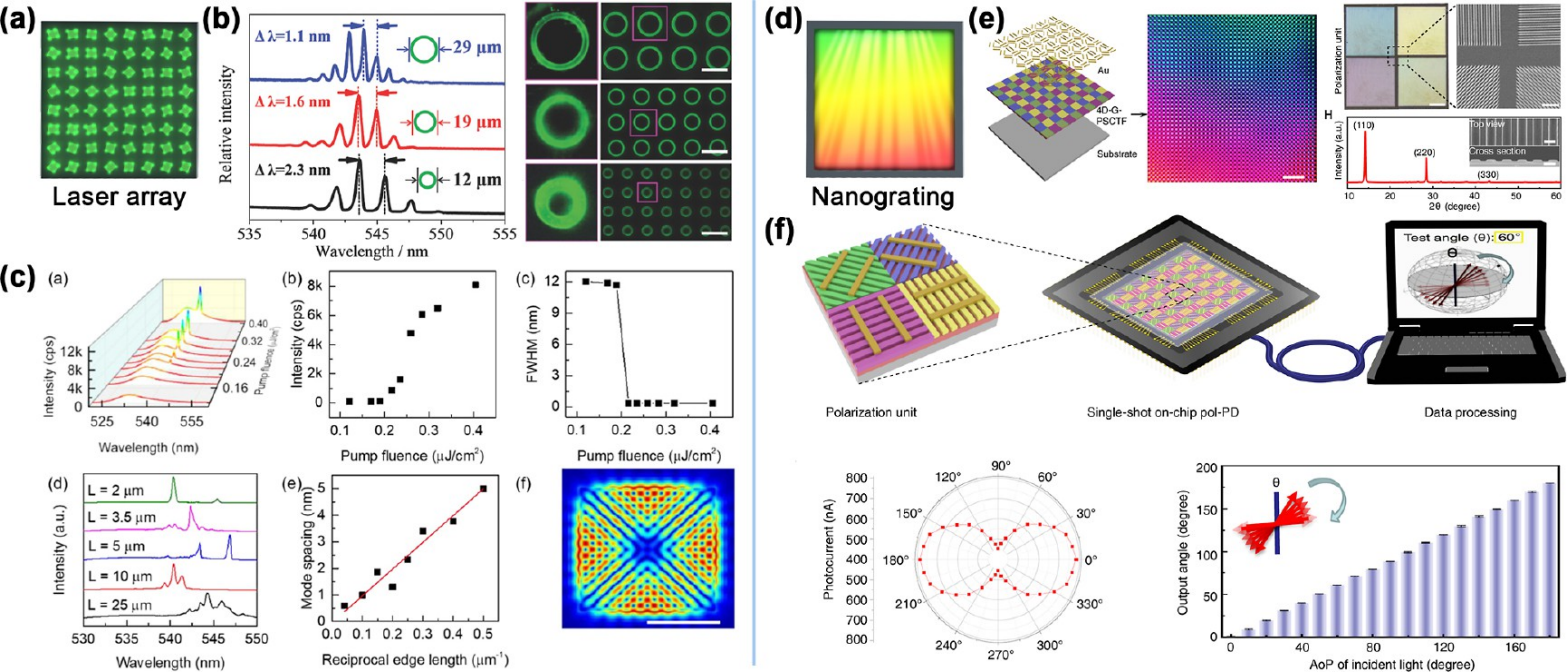
(a) Schematic diagram of the perovskite laser array. (b) μ-PL spectra and images collected from perovskite microrings with different diameters. (Reproduced with permission.^338^ Copyright 2018, Wiley-VCH.) (c) Multimode lasing spectra of five perovskite microplates with different edge lengths and the simulated electric field distribution inside the square perovskite cavity. (Reproduced with permission.^63^ Copyright 2021, Wiley-VCH.) (d) Schematic diagram of perovskite nanograting. (e,f) Microstructure and polarization photoelectric properties of four-directional grating array-capped perovskite single-crystal thin film. (Reproduced with permission.^55^ Copyright 2024, The American Association for the Advancement of Science.)

The construction of grating structures on a subwavelength scale can increase light extraction and the spontaneous emission probability, as illustrated in Figure 18d. Owing to the development of nanoimprinting technology, the PL of patterned films has been increased by two to three times, and the emission angle distribution has been concentrated within a clear angle range via optical resonance.^346^ Grating structures also help improve the performance of photodetectors^335^ and realize polarization imaging functions,^55^ as illustrated in Figure 18e,f. By rotating upper and lower gratings at a certain angle, a superimposed grating structure of a moiré lattice is generated, which enhances the absorption and reduces the reflection of incident light, significantly improving the performance of photodetectors.^351^ Periodically patterned perovskite nanostructures are used as emission layers to manufacture LEDs with approximately twice the radiance and a lower threshold than planar devices.^349^ In addition, the introduction of microstructures into the perovskite active layer of solar cells improves the photovoltaic performance, realizes nanophotonic light capture, and effectively suppresses carrier recombination.^343^

### 5.2 Patterned Optoelectronic Devices

Droplet array technology can provide high-precision control to ensure the orderly arrangement of perovskite materials on chips, thereby meeting the needs of high-resolution integrated optoelectronic devices.^380^ From the perspective of device construction, photodetectors can be easily fabricated through solution processing, as illustrated in Figure 19a. Taking a photoconductive detector as an example, only a perovskite light absorption layer between two electrodes needs to be formed to separate and drive the migration of photogenerated carriers under the driving of an electric field,^117^ as illustrated in Figure 19b. In addition, this device has low dependence on material interface quality and complex stacking, so preparation via droplet array technology is more suitable.^54^ For example, inkjet printing and the wettability-assisted surface limitation method are used to accurately distribute the perovskite precursor solution at specified positions on the substrate to ensure efficient construction of lateral structure photodetectors^57,117,152,158,166^ and vertical structure photodetectors,^235,236,381^ providing possibilities for high-resolution imaging. In maskless, noncontact inkjet printing, the composition of the perovskite precursor solution is adjusted to construct pixelated photodetector arrays with perovskite materials of different band gaps to achieve a multispectral response.^140,189^ Additionally, imprinting and template confinement methods help in the construction of low-dimensional perovskite photodetectors.^216,221,293^ Owing to their strong anisotropy, PNWs can efficiently detect polarized light. Figure 19c shows that the maximum photocurrent appears in the direction parallel to the axis of the one-dimensional array, whereas the minimum photocurrent appears when the polarization angle is rotated 90°, thereby generating polarization anisotropy.^222^ Consequently, perovskite devices based on photoelectric sensing have demonstrated remarkable potential in innovative applications such as image sensors,^6,382^ health monitoring,^383,384^ and human–machine interaction.^117,385^ These applications not only broaden the application scope of perovskite materials but also provide new possibilities for their development in wearable devices and intelligent interactive systems.

**Figure 19:**
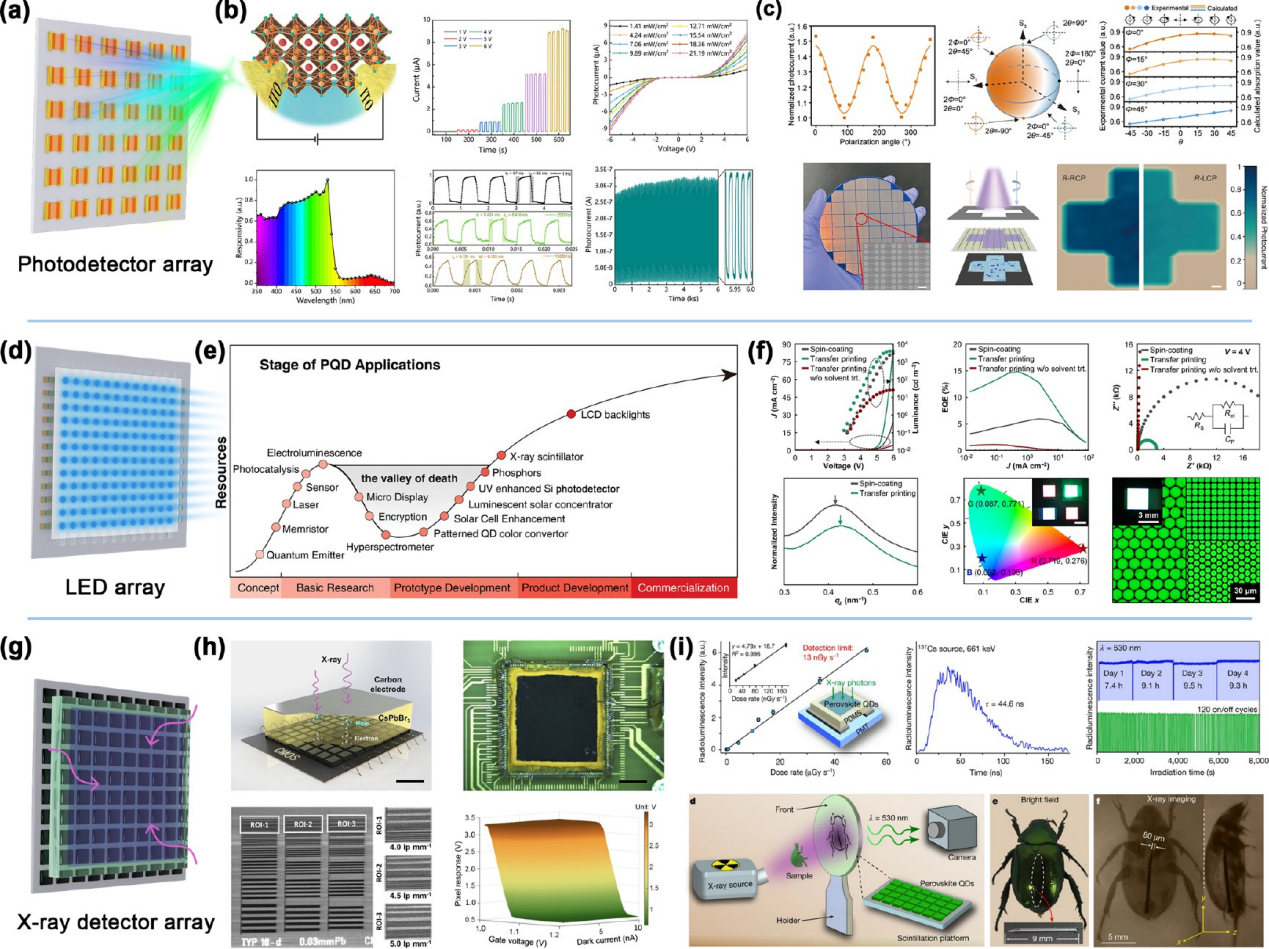
(a) Schematic diagram of the perovskite photodetector array. (b) Photodetection performance of an individual perovskite photodetector on a glass substrate. (Reproduced with permission.^117^ Copyright 2022, The Authors, published by Springer Nature.) (c) Full-Stokes polarimeter and imaging of chiral 3D perovskite microwire arrays. (Reproduced with permission.^222^ Copyright 2024, American Chemical Society.) (d) Schematic diagram of the perovskite LED array. (e) Development stages of applications based on in situ fabricated perovskite QDs. (Reproduced with permission.^386^ Copyright 2024, Wiley-VCH.) (f) EL characteristics of transfer-printed PeLEDs. (Reproduced with permission.^359^ Copyright 2022, The American Association for the Advancement of Science.) (g) Schematic diagram of the perovskite X-ray detector array. (h) Illustration of the X-ray detector structure and X-ray responses of the screen-printed perovskite CMOS array. (Reproduced with permission.^307^ Copyright 2024, The Authors, published by Springer Nature.) (i) Ultrasensitive X-ray sensing and radiography using CsPbBr_3_ nanocrystals. (Reproduced with permission.^387^ Copyright 2018, Springer Nature.)

Perovskite LEDs consist of perovskite emitters sandwiched between hole transport and electron transport layers, as shown in Figure 19d. Under a forward bias, holes and electrons are injected into the perovskite emitter layer, forming electron–hole pairs, which then radiatively recombine to convert electrical energy into light emission.^388^ As illustrated in Figure 19e, based on an evaluation of the maturity of their technology and material development, perovskite LEDs that meet the needs of miniaturization and flexibility have extremely high prospects for industrial development.^386^ By combining droplet technology with advanced printing technology, we can overcome the limitations of traditional manufacturing processes for perovskite LEDs, laying the foundation for efficient and low-cost commercial applications. These LEDs are expected to play an important role in flexible displays and high-color saturation imaging.^388^ The construction of perovskite LEDs requires careful consideration of the processing technology so that the processing does not damage the multilayer structure or underlying circuits.^389^ Therefore, the use of nondestructive sequential patterning methods such as inkjet printing and transfer printing to manufacture multicolor perovskite LEDs is promising.^195^ For example, during inkjet printing, solvent engineering and the evaporation dynamics are used to suppress the coffee ring effect to prepare multicolor perovskite LEDs^154,188^ and QLEDs.^160^ The transfer printing, as shown in Figure 19f, using viscoelastic PDMS stamps offers the possibility of achieving full-color LEDs.^359^ This process does not use wet chemicals, avoids the solvent orthogonality problem, and prevents cross-contamination of pixels of different colors.

The exploration of perovskites’ potential in the field of X-rays has long been a crucial area of research,^387,390,391^ as shown in Figure 19g. The successful integration of perovskite materials with thin-film transistor arrays^173,392,393^ complementary metal–oxide–semiconductors (CMOSs)^307,394^ lays the foundation for high-resolution, real-time, and multipixel X-ray imaging,^395^ as illustrated in Figure 19h,i. Furthermore, the exploration of perovskite devices by researchers has not ceased; they are actively extending this technology to other cutting-edge fields, including solar cells,^396^ memristors,^397^ humidity sensors,^398,399^ and gas sensors.^400,401^ Exploration in these fields not only deepens the understanding of the properties of perovskite materials but may also lead to a series of novel technologies and applications.

## 6 Conclusion and Outlook

Droplet array technology has emerged as a core driving force in the fabrication of perovskite optoelectronic device arrays, demonstrating unique advantages in enabling low-cost, large-scale manufacturing. This review systematically reviews the key role of droplet technology in arraying perovskite materials, focusing on the physical properties of droplets, the array generation mechanism, and its influence on the crystallization and patterning of perovskite materials. Furthermore, this review emphasizes the relationship between the fabrication method, material structure, and device performance. For instance, the design of physical constraints, such as microchannels or surface confinement, enables precise positioning and orientation control of perovskite arrays. Such controlled fabrication lays a crucial foundation for achieving superior optoelectronic properties and uniform device performance across arrays.

However, several urgent problems need to be solved in the industrialization of perovskite materials and devices. The environmental sensitivity and ion migration problems of perovskites can degrade device performance.^402,403^ Packaging technology development, solvent engineering, and interface engineering have been used to address various challenges related to stability, thereby ensuring good device performance and service life.^404,405^ In addition, the potential toxicity of the common lead component in perovskites has necessitated the development of lead-free compounds.^406–408^ The high demand for perovskites in the field of solar cells makes the toxicity of solvents a challenge. For example, the solvent DMF of precursor solutions and the solvents hexane and toluene of QD dispersions have adverse effects on the environment and human health, so the exploration of environmentally friendly solvent systems suitable for industrial production is still ongoing.^409^

Future research on the use of a solution-based patterning method in perovskite material synthesis will focus on the self-assembly mechanisms, interface properties, and solute distribution on crystal morphology. Particularly for polycrystalline perovskite thin films, controlling crystalline orientation during crystallization can modify the charge transport properties of the film.^410,411^ Therefore, patterning techniques for the oriented growth of both polycrystalline films and single crystals represent promising research directions for enhancing device performance.

Combined with developments in artificial intelligence, material genome engineering will further accelerate the process of material discovery and development, and through the close integration of high-throughput experiments and data mining, the performance of arrayed materials will be efficiently screened and optimized. Expanding droplet array technology to advanced materials such as photonic crystals, various QDs, and organic optoelectronics will promote the development of high-performance devices.

## Vocabulary

Wettability: The tendency of a liquid to spread over or retract from a solid surface, quantified by the contact angle, which is governed by the balance of interfacial tensions at the solid–liquid–gas triple phase.
Capillary force: The net force arising from the Laplace pressure difference across a curved liquid–air interface (meniscus), driven by surface tension and modulated by the solid’s wettability. It acts to draw liquid into narrow channels or porous media.
Capillary flow: The compensatory internal liquid movement within an evaporating droplet, driven by an evaporation flux difference between the pinned contact line and the droplet center, which dominates solute transport to the triple-phase contact line.
Crystallization: The process whereby solute atoms or molecules precipitate from a supersaturated solution and arrange into a regular, periodic lattice structure. High-quality crystalline films are characterized by continuity, an absence of pinholes, and tightly bonded grain boundaries.
Nanocrystals: Crystalline materials with at least one dimension in the approximate range of 1 to 100 nm, exhibiting physicochemical properties distinct from bulk materials due to pronounced surface and quantum size effects.
Quantum dots: Nanocrystals whose size is below the exciton Bohr radius, confining charge carriers in three dimensions and resulting in size-tunable optical and electronic properties due to discrete energy levels.
Microstructure template: A prepatterned surface with specific topography fabricated via techniques like lithography or etching; its physical and chemical properties spatially confine droplet dynamics to guide the crystallization of materials into designed patterns.
